# Supplementary figures and images for: Identification of potential functional peptides involved in demyelinating injury in the central nervous system
Source: PeerJ. 2023 Aug 21;11:e15846. doi: 10.7717/peerj.15846 (PMC10448882; doi:10.7717/peerj.15846)

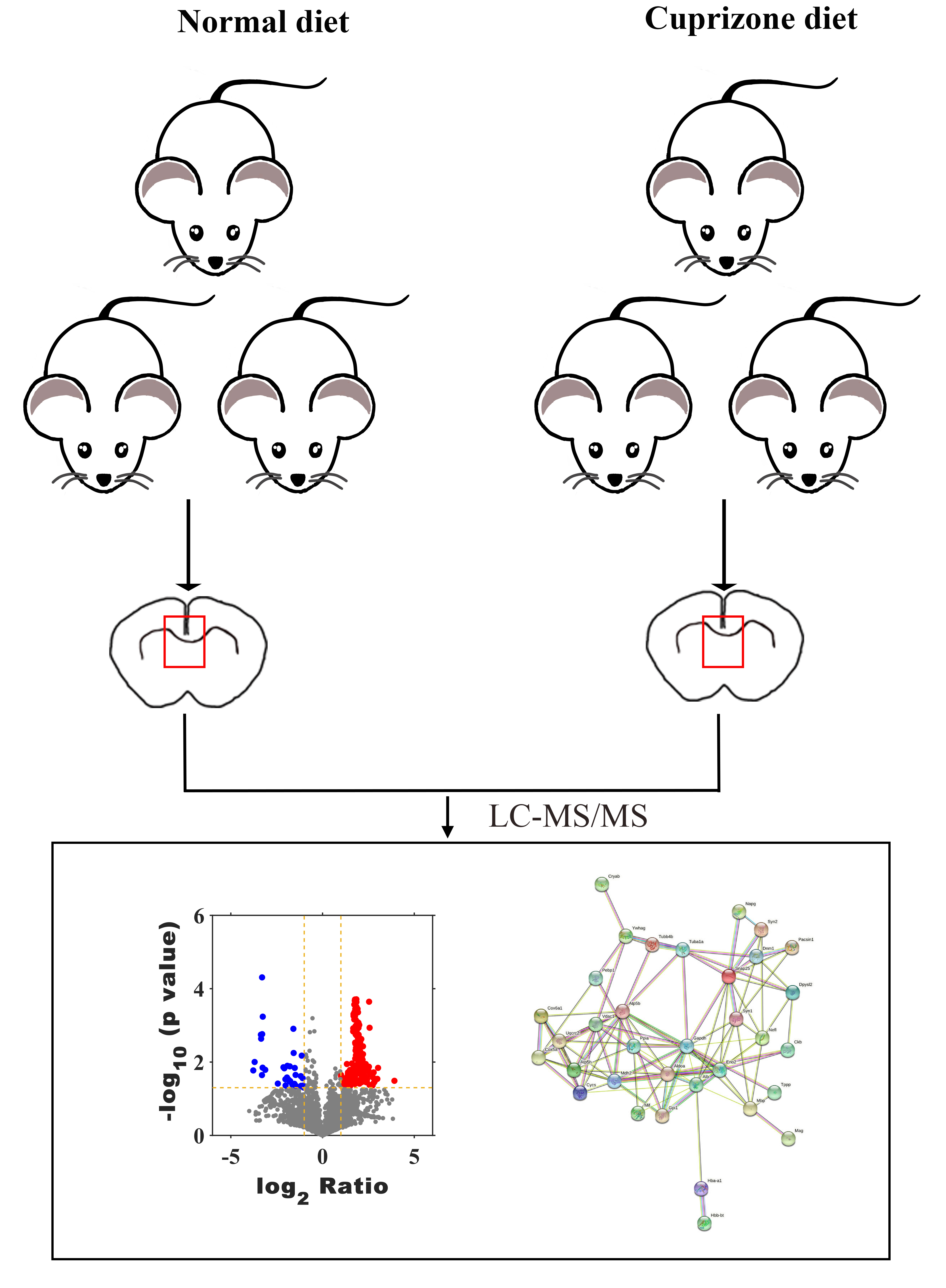

Supplement: Supplemental Information 1 [file peerj-11-15846-s001.jpg]

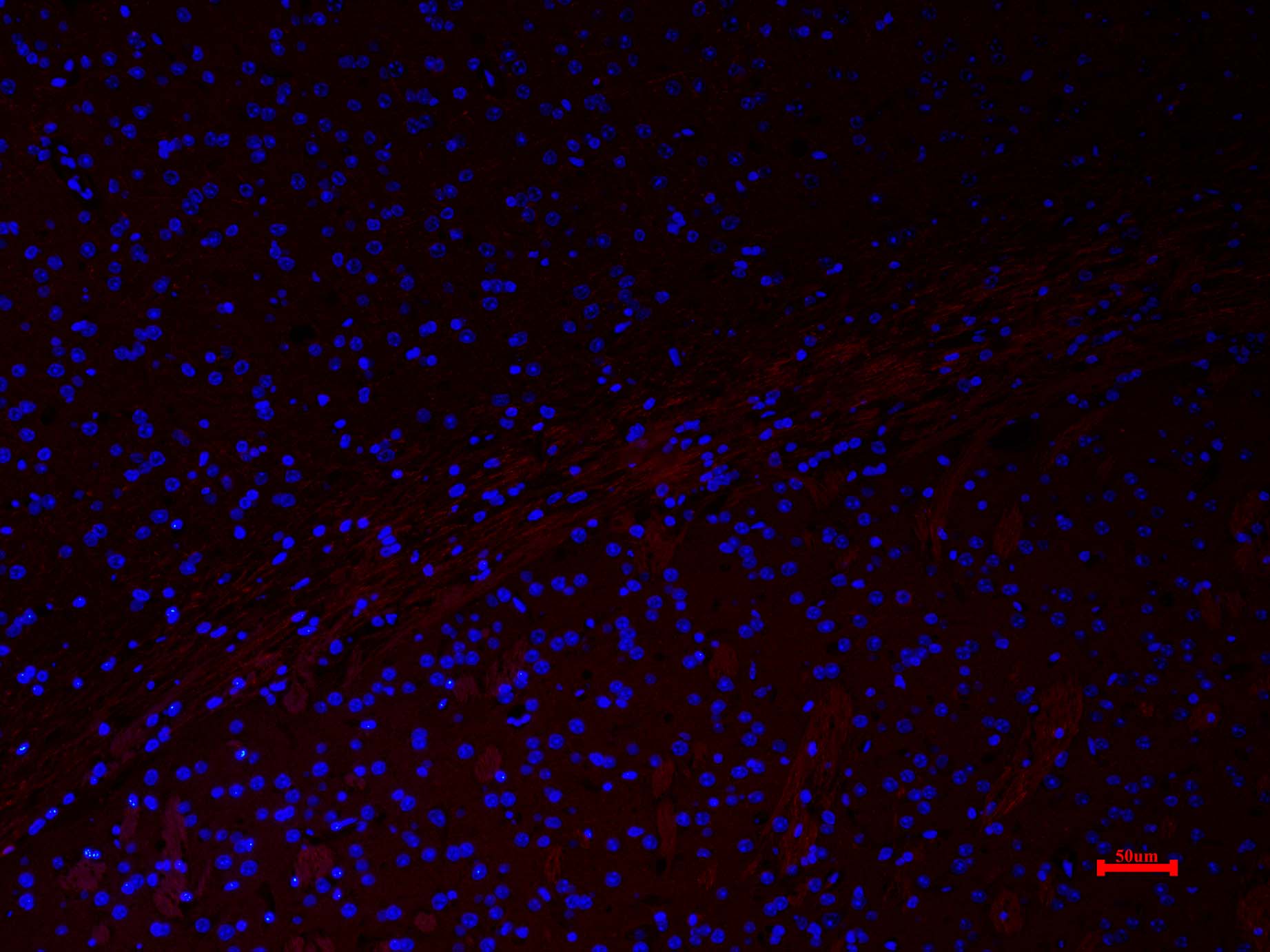

Supplement: Supplemental Information 3 [file peerj-11-15846-s003.zip › Supplemental Files 1/MAG/CPZ-MERGE-200├ù.jpg]

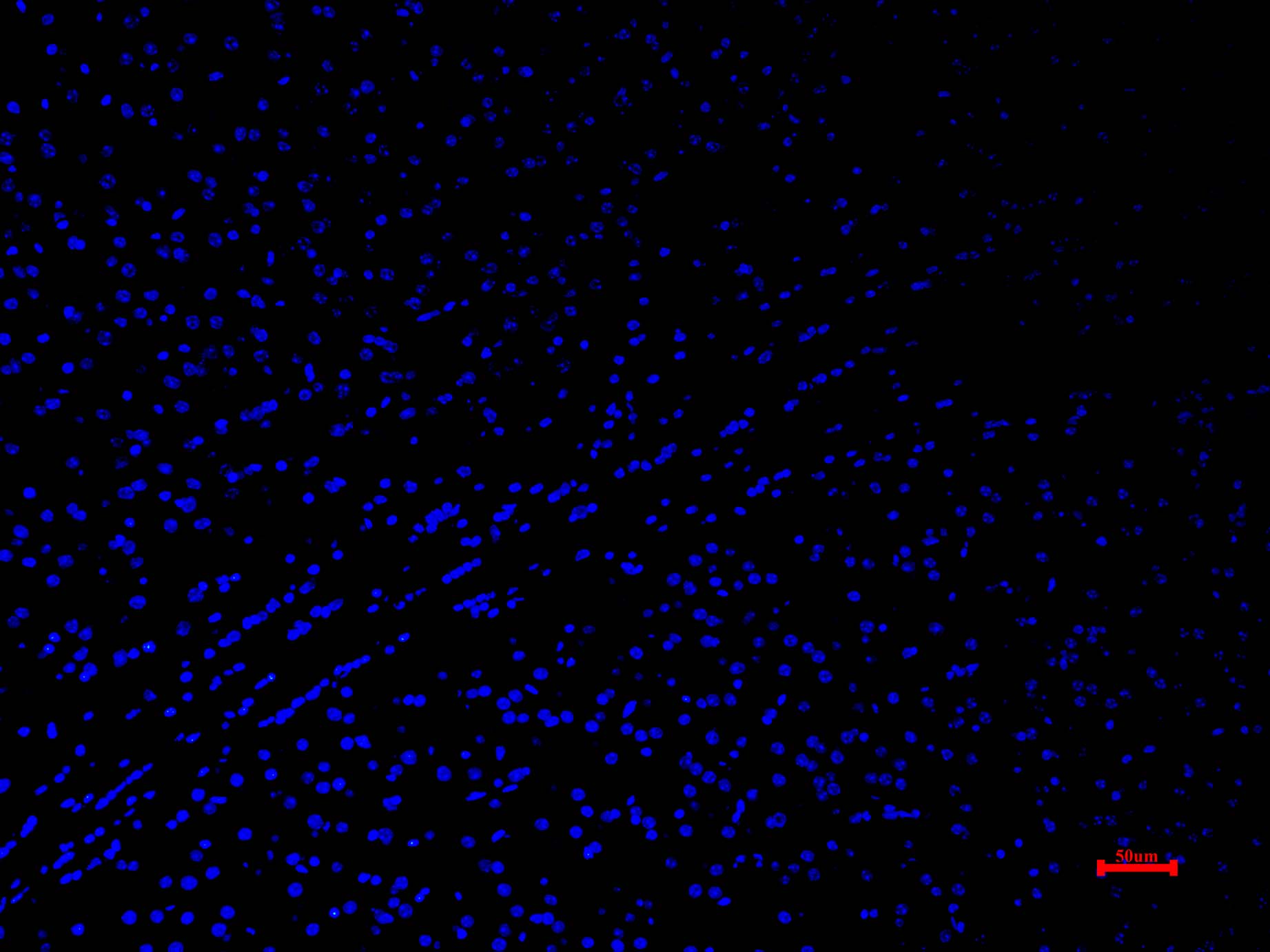

Supplement: Supplemental Information 3 [file peerj-11-15846-s003.zip › Supplemental Files 1/MAG/Control-DAPI-200├ù.jpg]

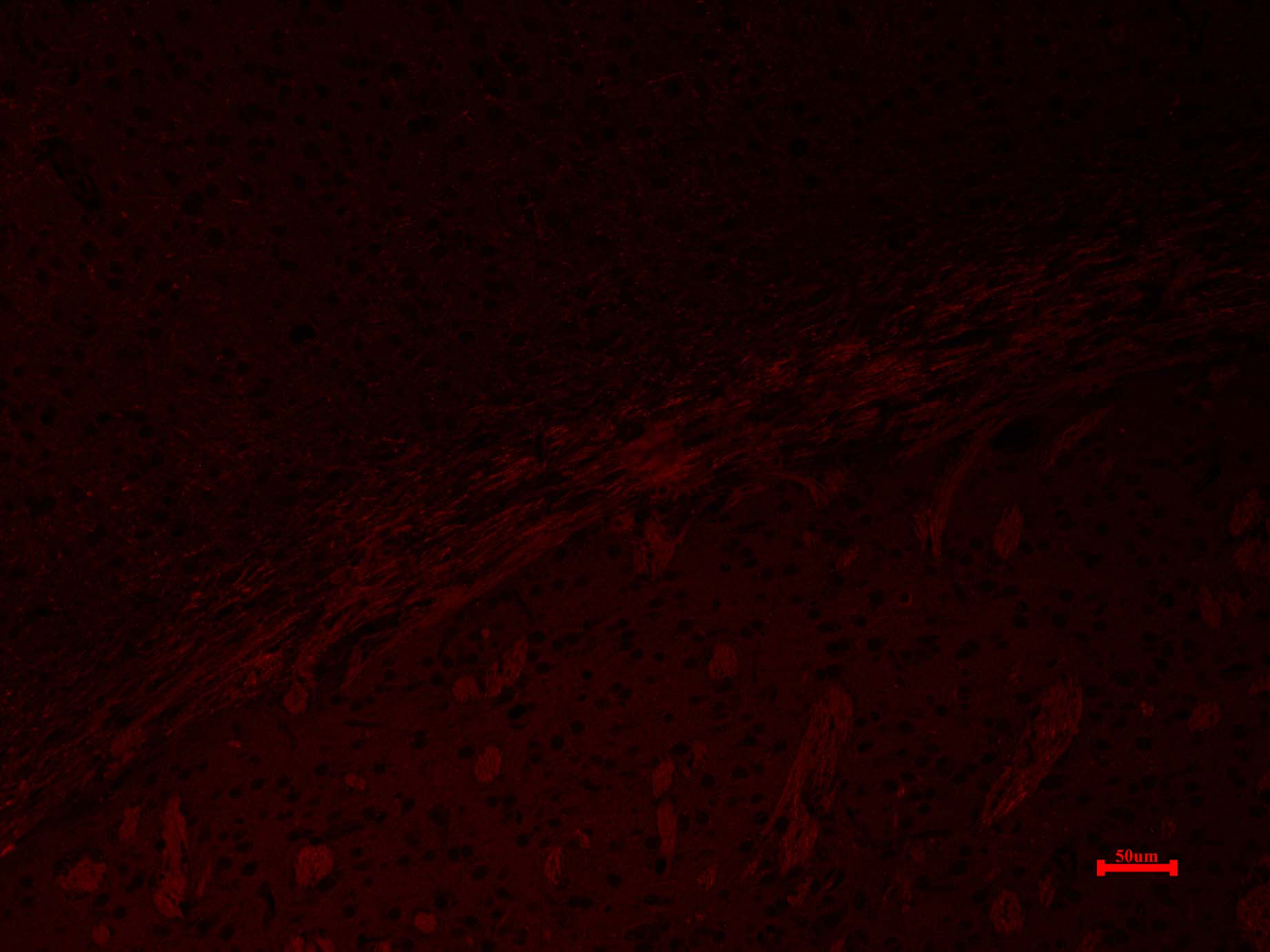

Supplement: Supplemental Information 3 [file peerj-11-15846-s003.zip › Supplemental Files 1/MAG/CPZ-200├ù.jpg]

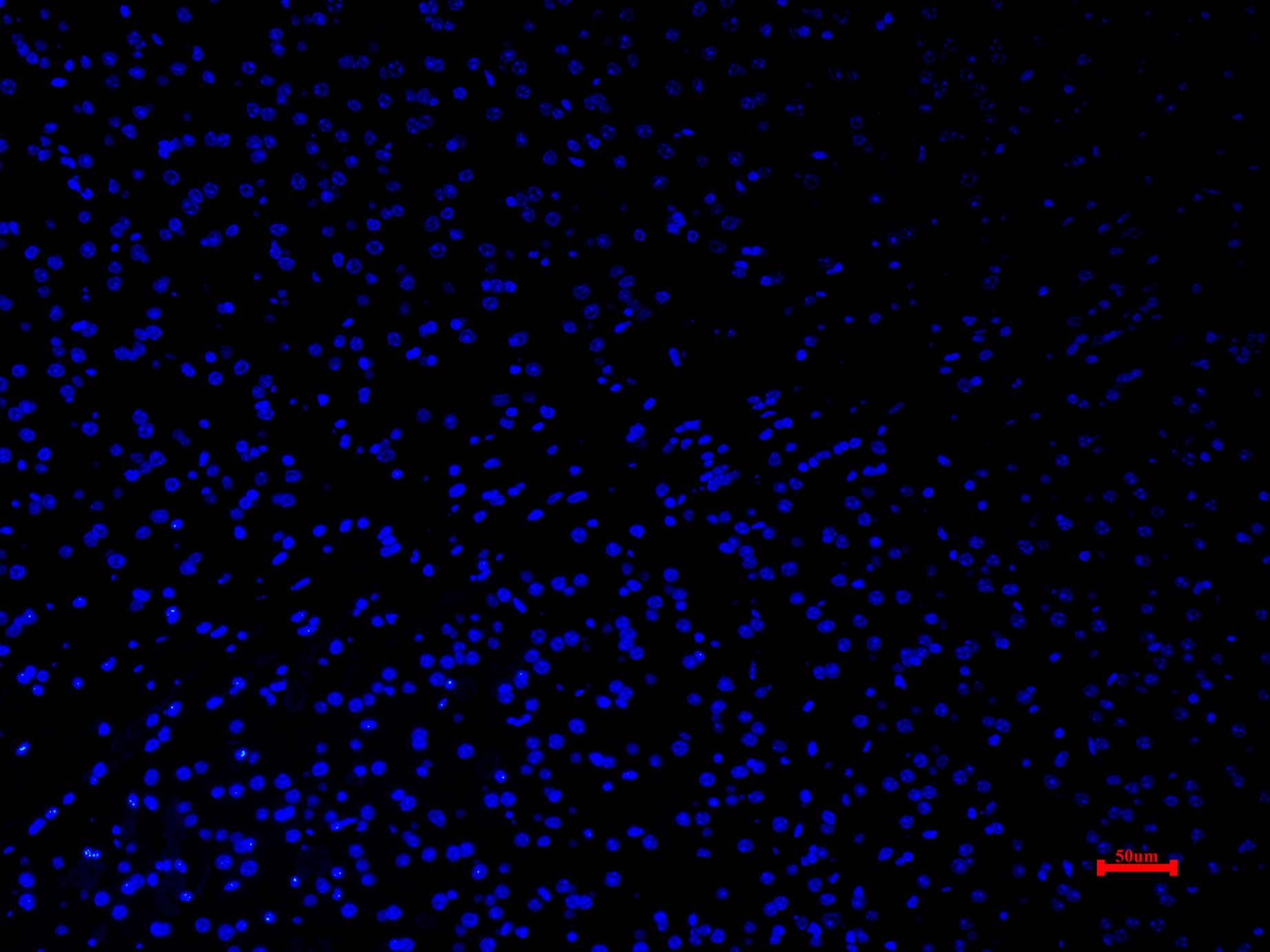

Supplement: Supplemental Information 3 [file peerj-11-15846-s003.zip › Supplemental Files 1/MAG/CPZ-DAPI-200├ù.jpg]

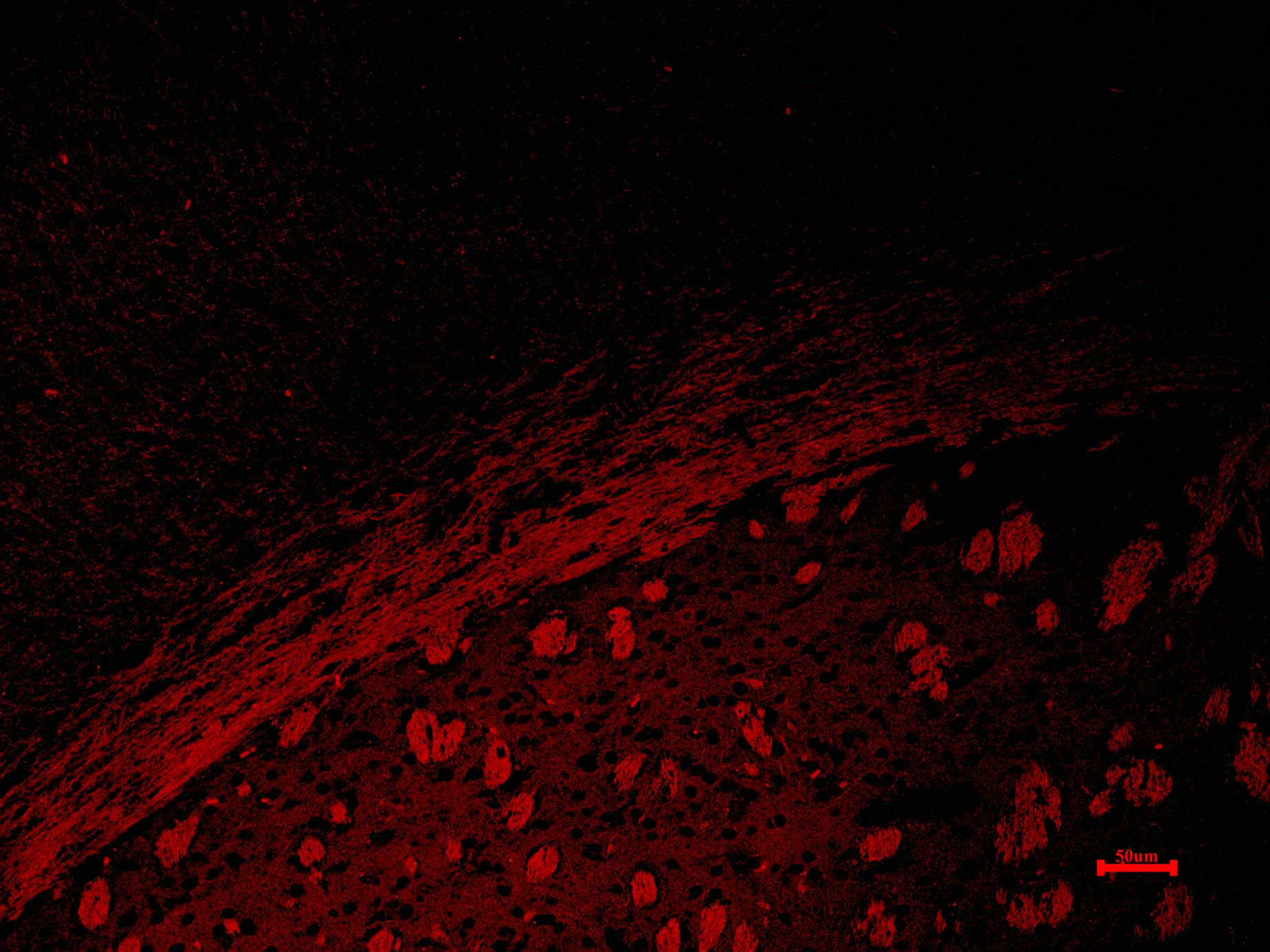

Supplement: Supplemental Information 3 [file peerj-11-15846-s003.zip › Supplemental Files 1/MAG/Control-200├ù.jpg]

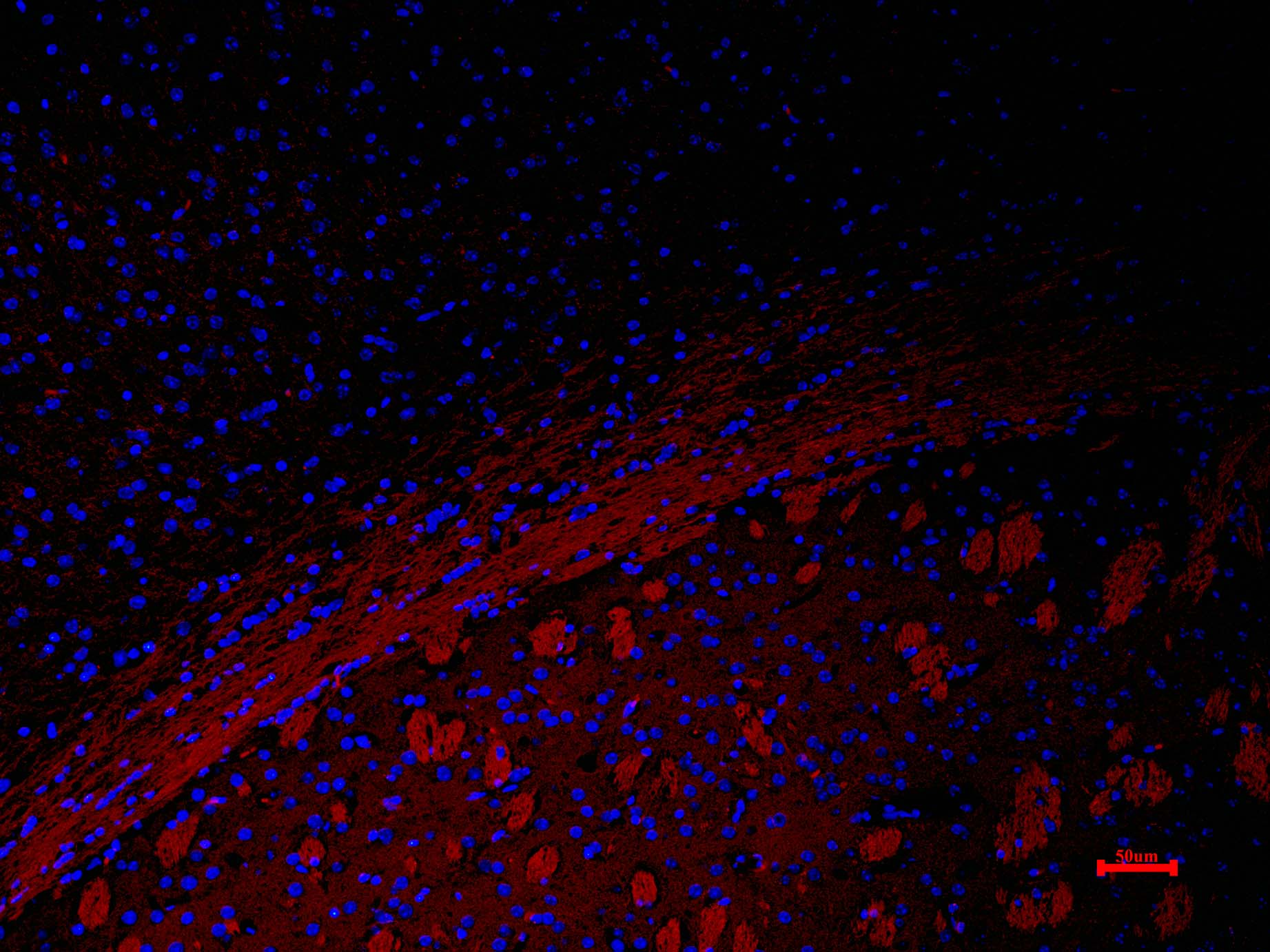

Supplement: Supplemental Information 3 [file peerj-11-15846-s003.zip › Supplemental Files 1/MAG/Control-MERGE-200├ù.jpg]

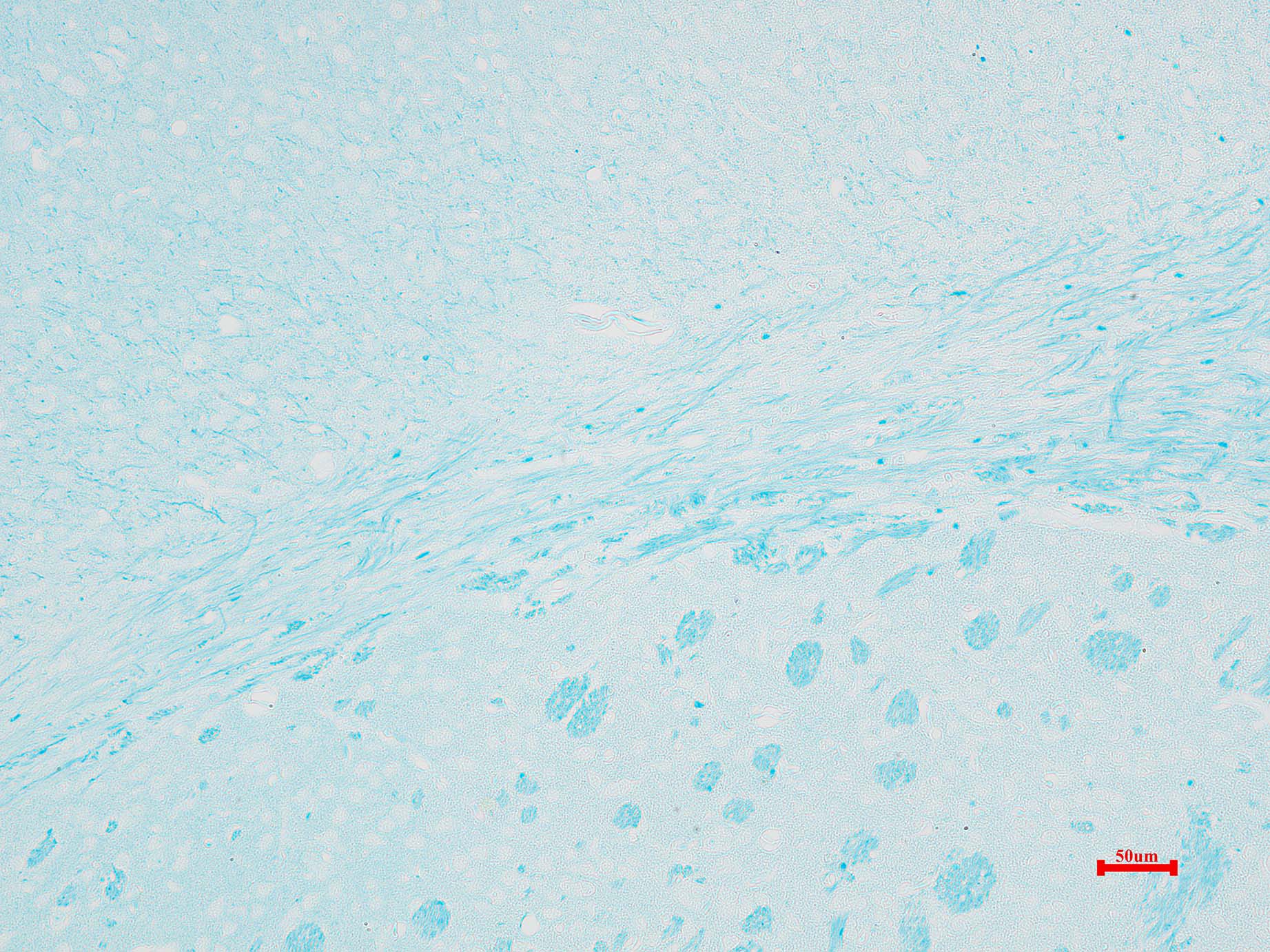

Supplement: Supplemental Information 3 [file peerj-11-15846-s003.zip › Supplemental Files 1/LFB/LFB-CPZ-200X-2.jpg]

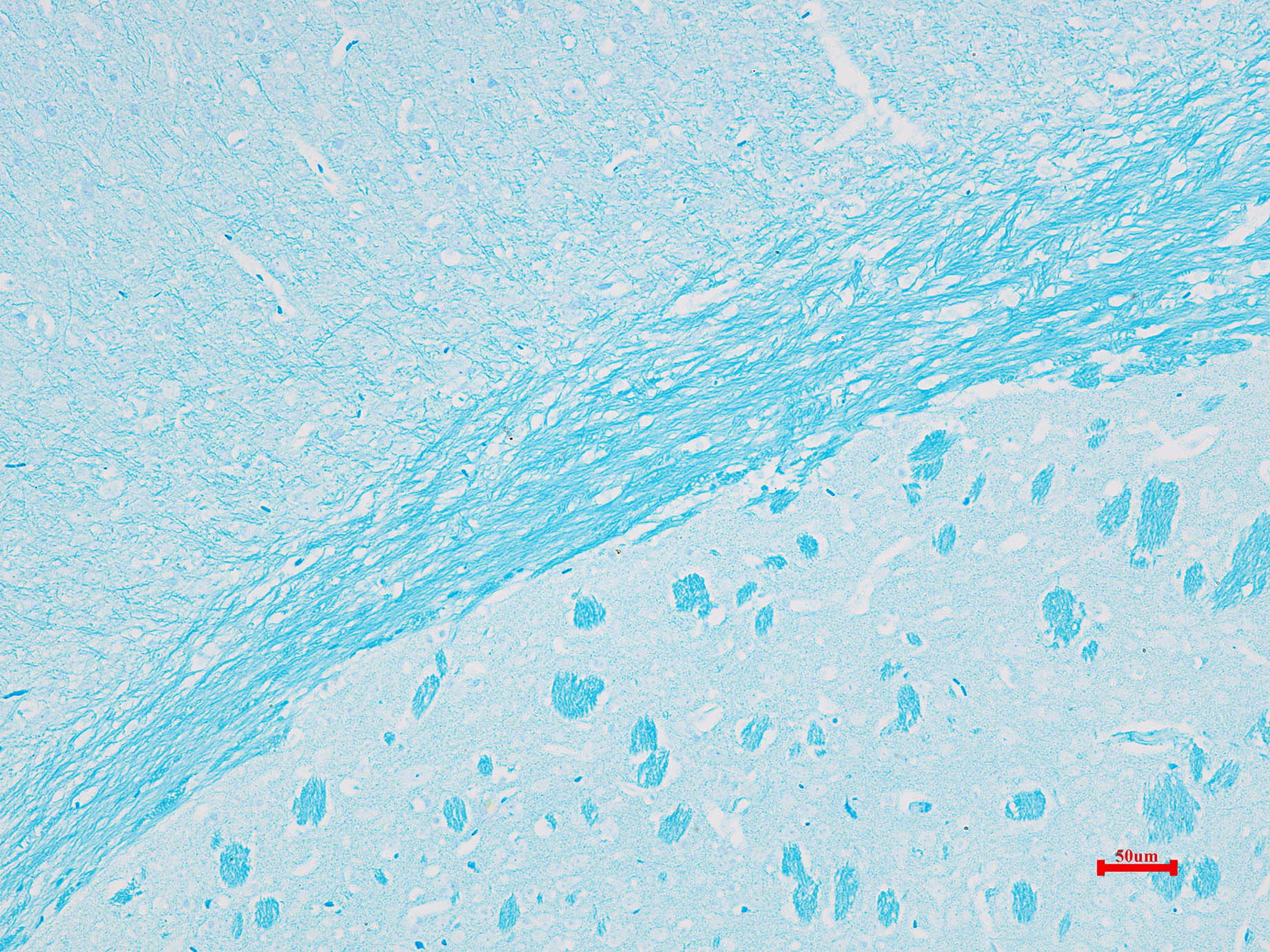

Supplement: Supplemental Information 3 [file peerj-11-15846-s003.zip › Supplemental Files 1/LFB/LFB-control-200X-2.jpg]

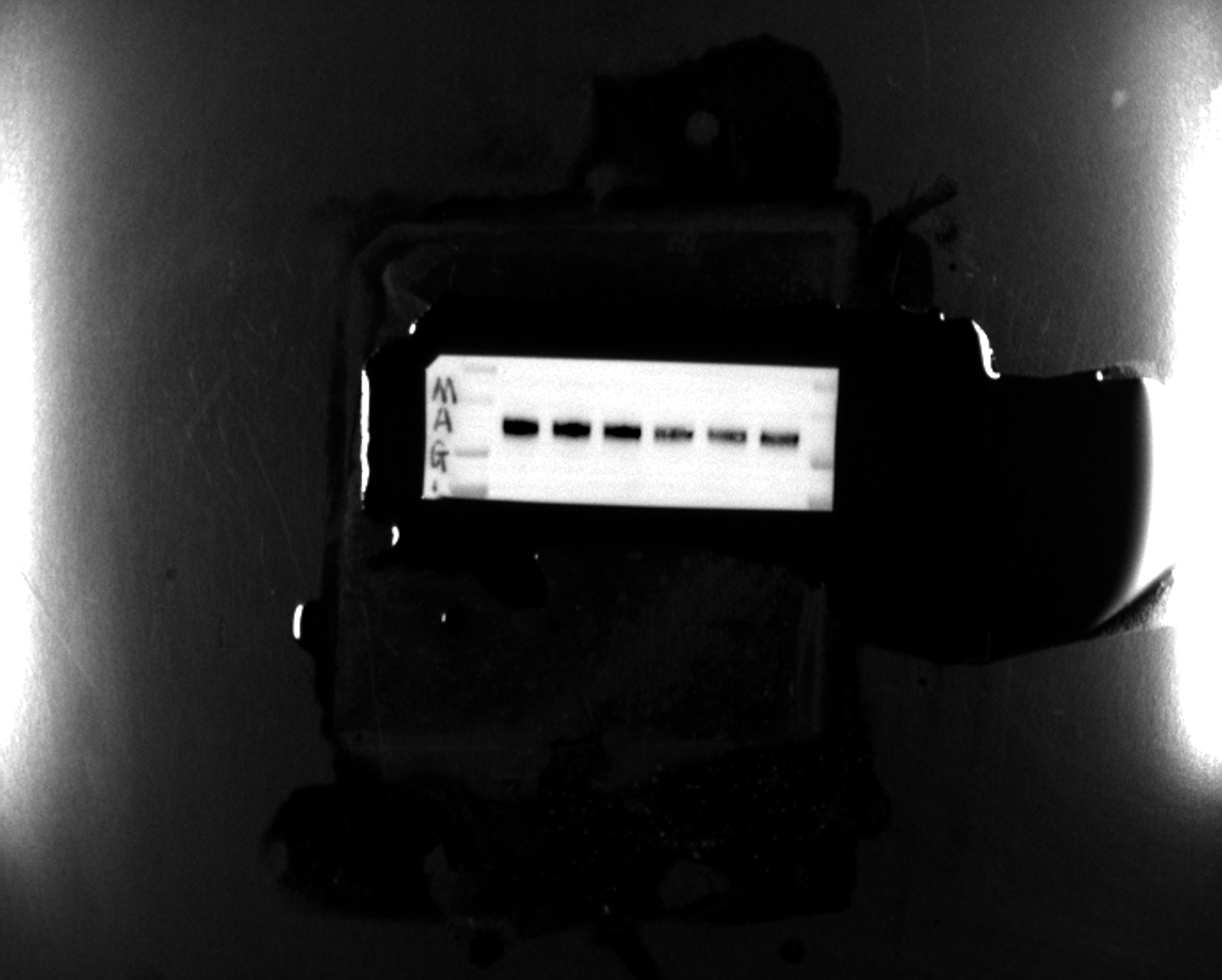

Supplement: Supplemental Information 3 [file peerj-11-15846-s003.zip › Supplemental Files 1/WB/MAG M.Tif]

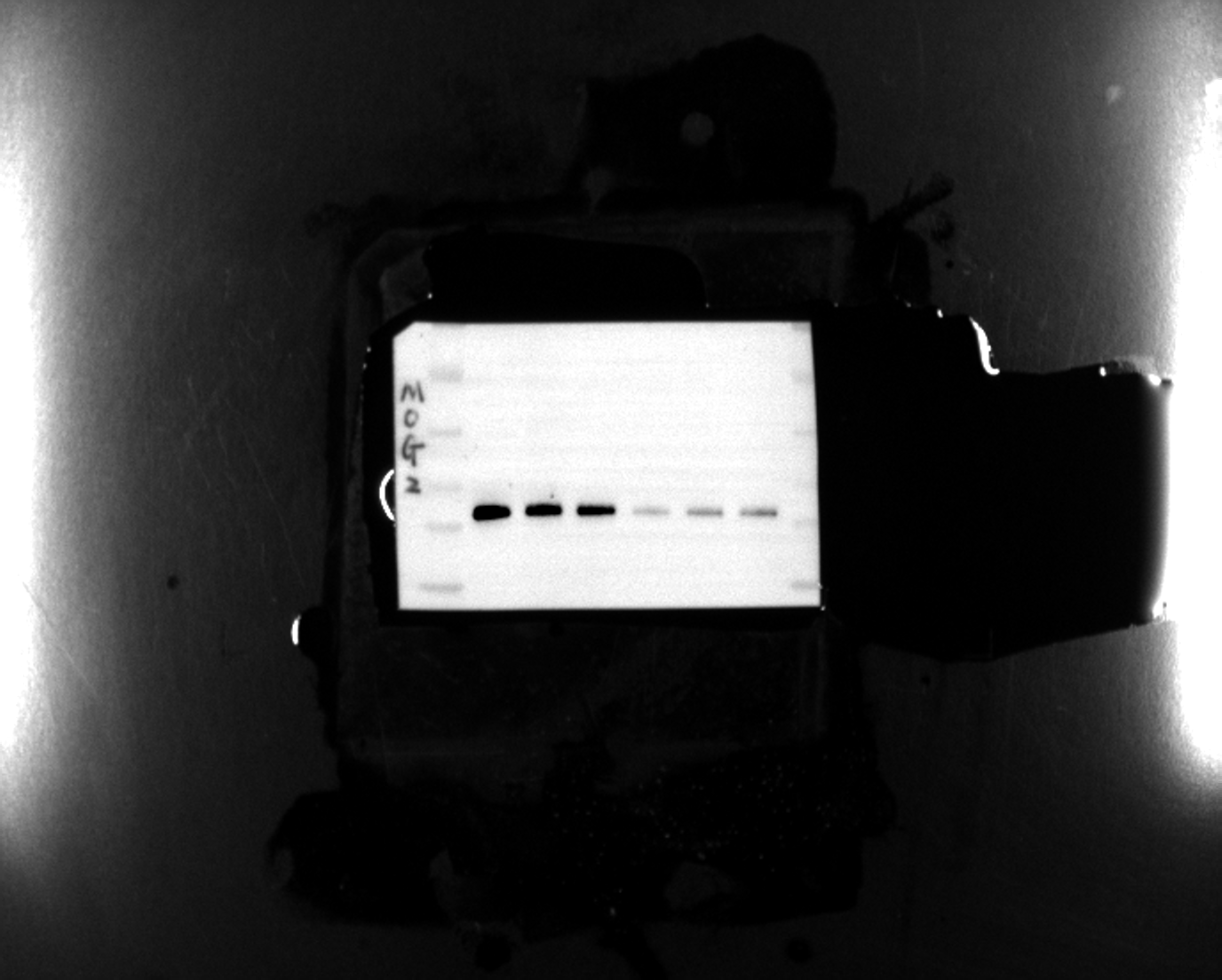

Supplement: Supplemental Information 3 [file peerj-11-15846-s003.zip › Supplemental Files 1/WB/MOG M.Tif]

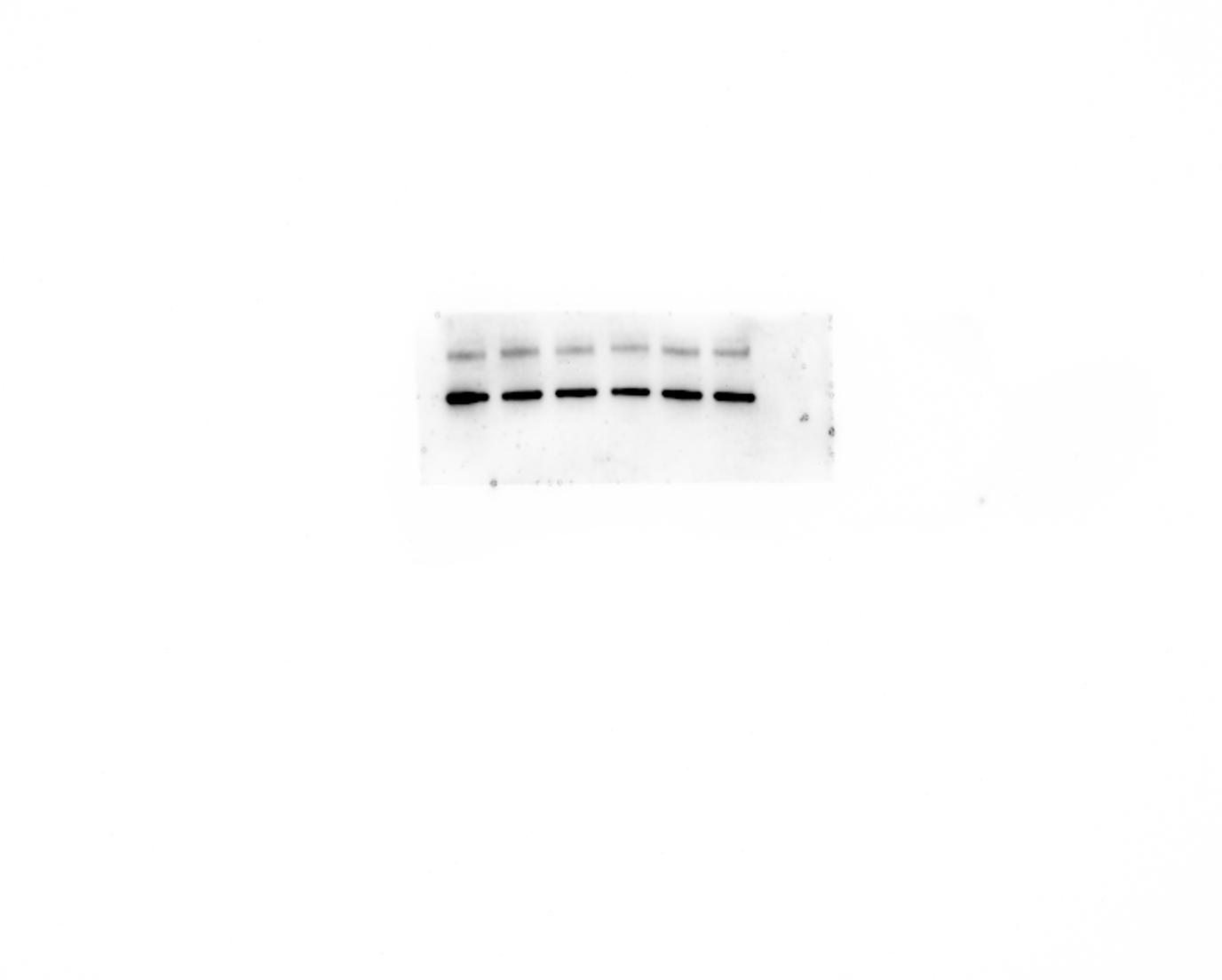

Supplement: Supplemental Information 3 [file peerj-11-15846-s003.zip › Supplemental Files 1/WB/2-b-actin 10M.Tif]

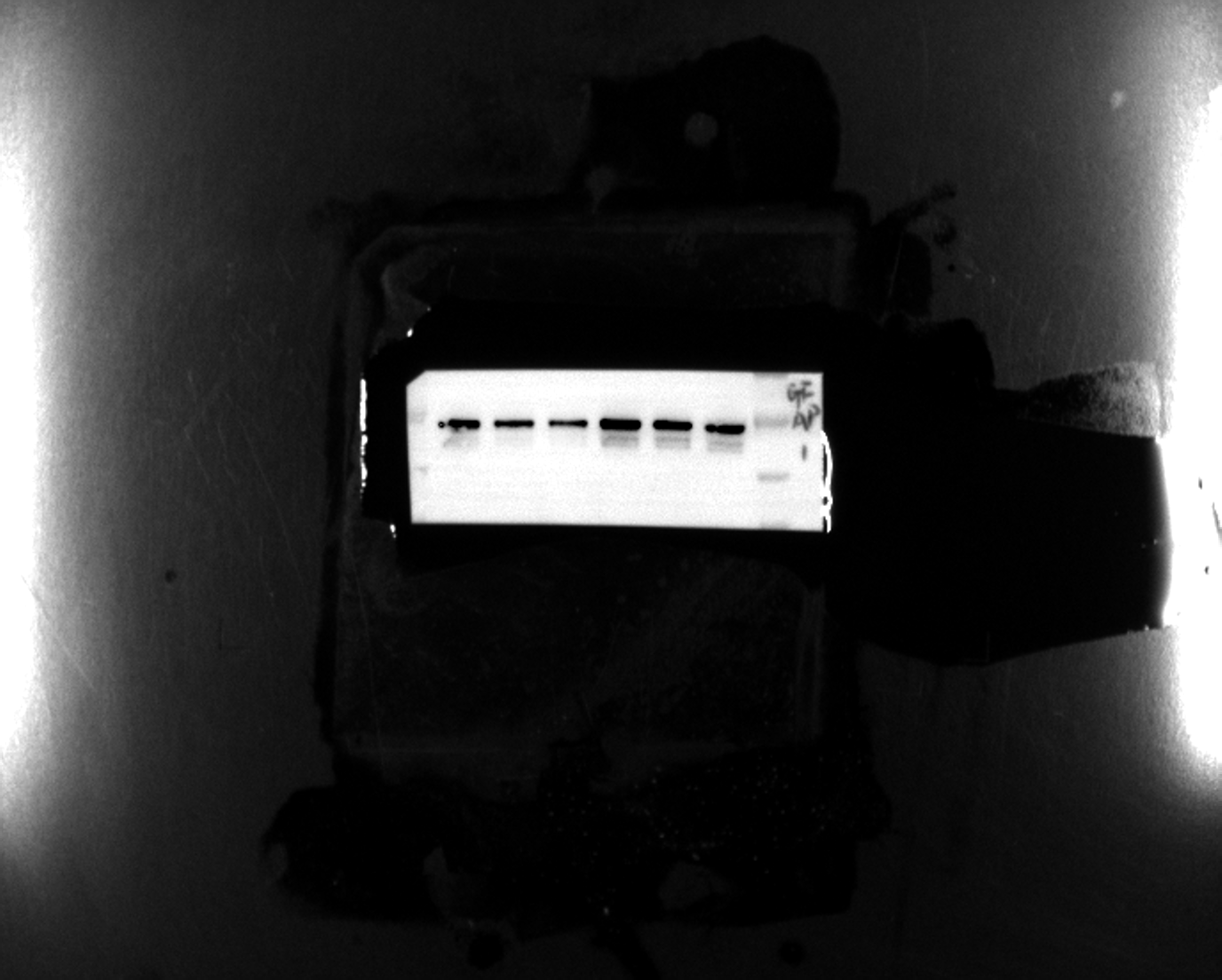

Supplement: Supplemental Information 3 [file peerj-11-15846-s003.zip › Supplemental Files 1/WB/GFAP M-2.Tif]

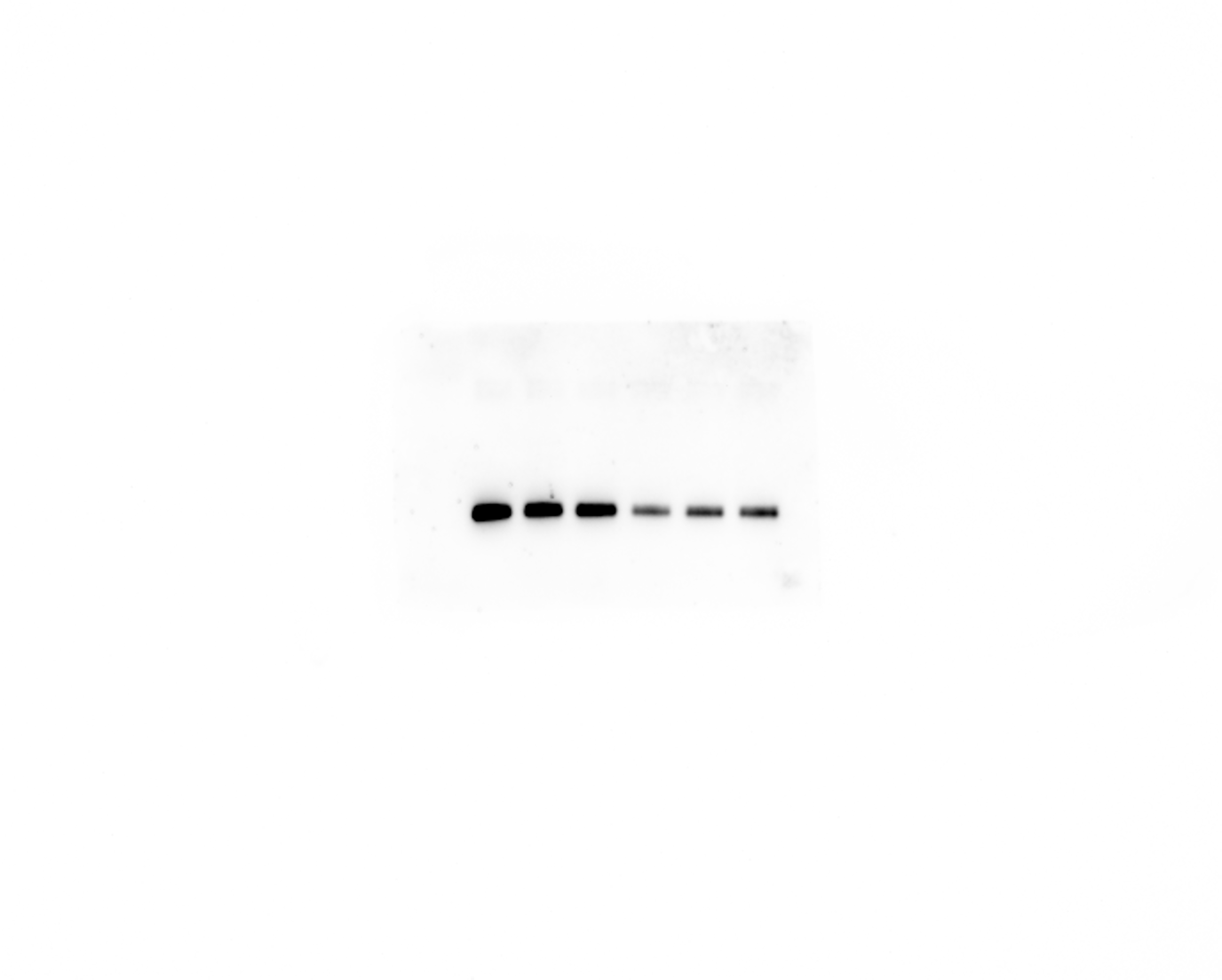

Supplement: Supplemental Information 3 [file peerj-11-15846-s003.zip › Supplemental Files 1/WB/MOG 5M.Tif]

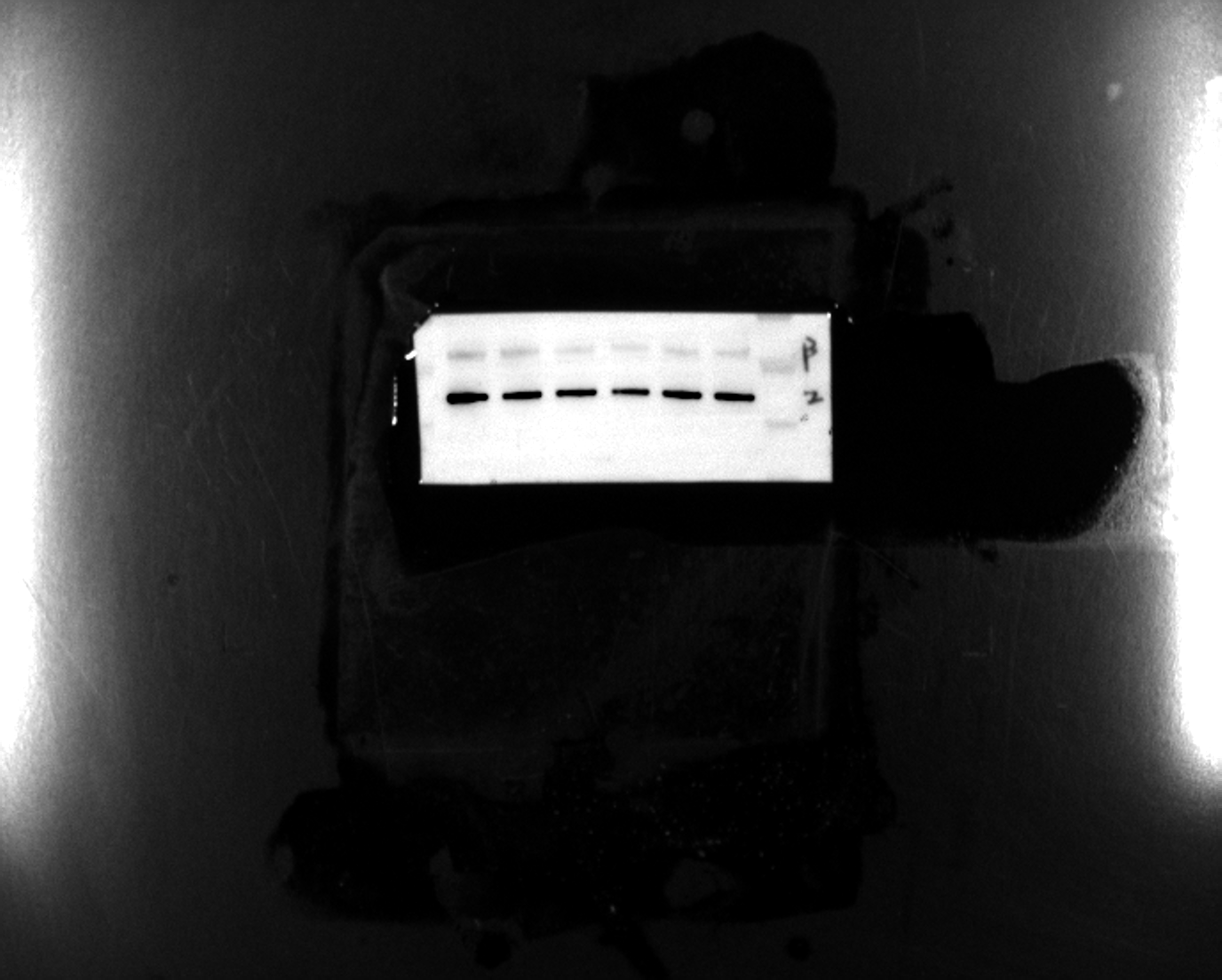

Supplement: Supplemental Information 3 [file peerj-11-15846-s003.zip › Supplemental Files 1/WB/2-b-actin M.Tif]

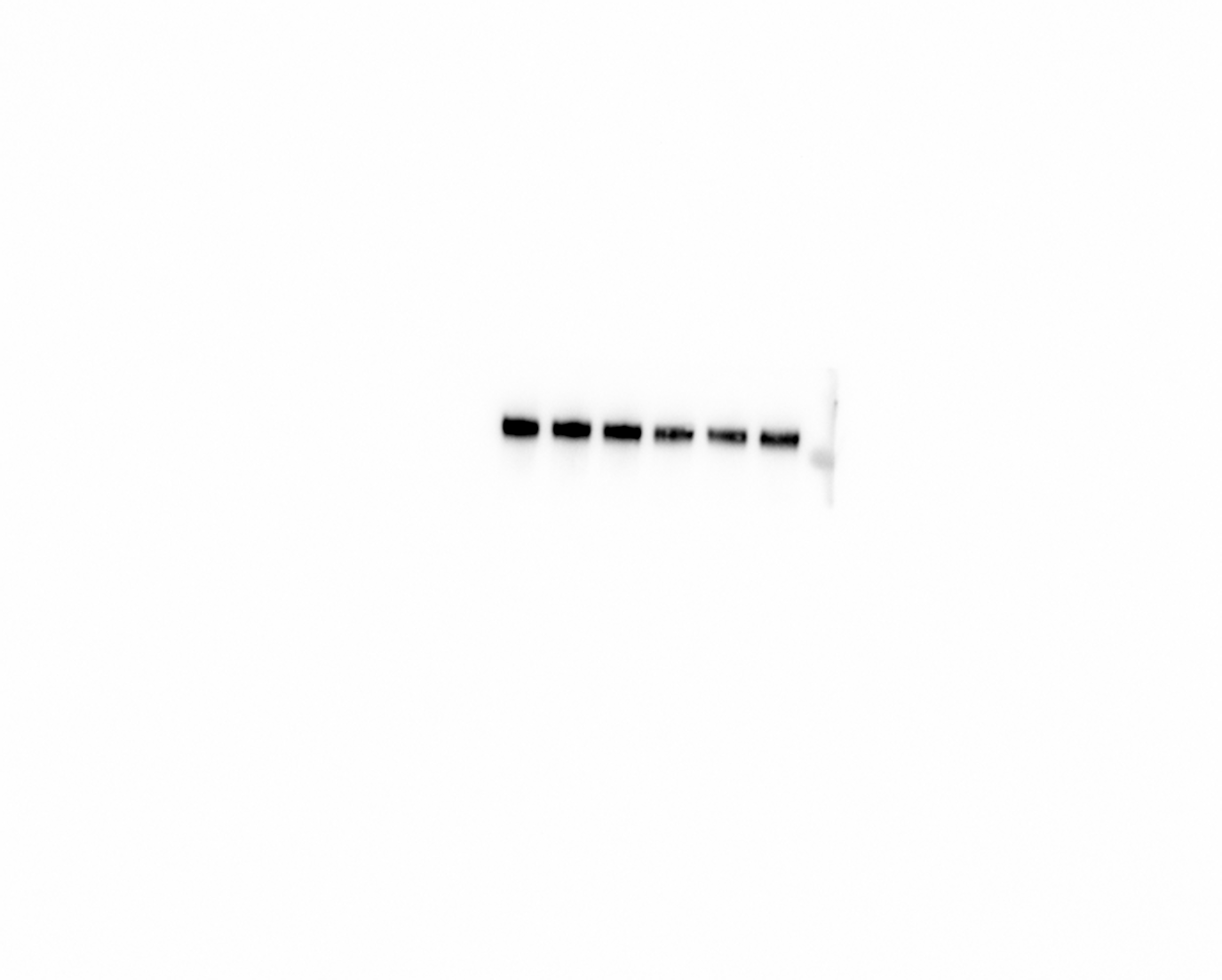

Supplement: Supplemental Information 3 [file peerj-11-15846-s003.zip › Supplemental Files 1/WB/MAG 5S.Tif]

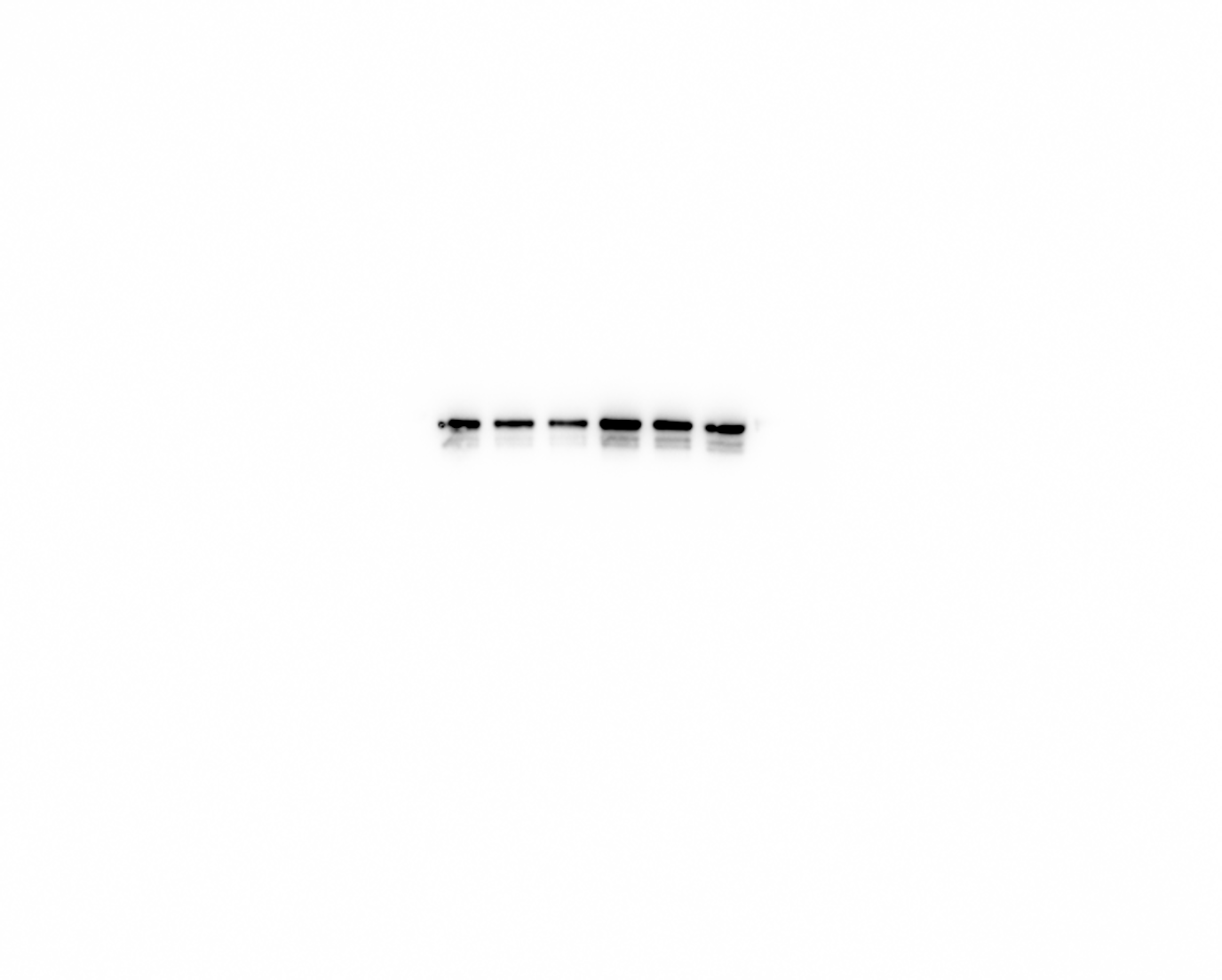

Supplement: Supplemental Information 3 [file peerj-11-15846-s003.zip › Supplemental Files 1/WB/GFAP 5S.Tif]

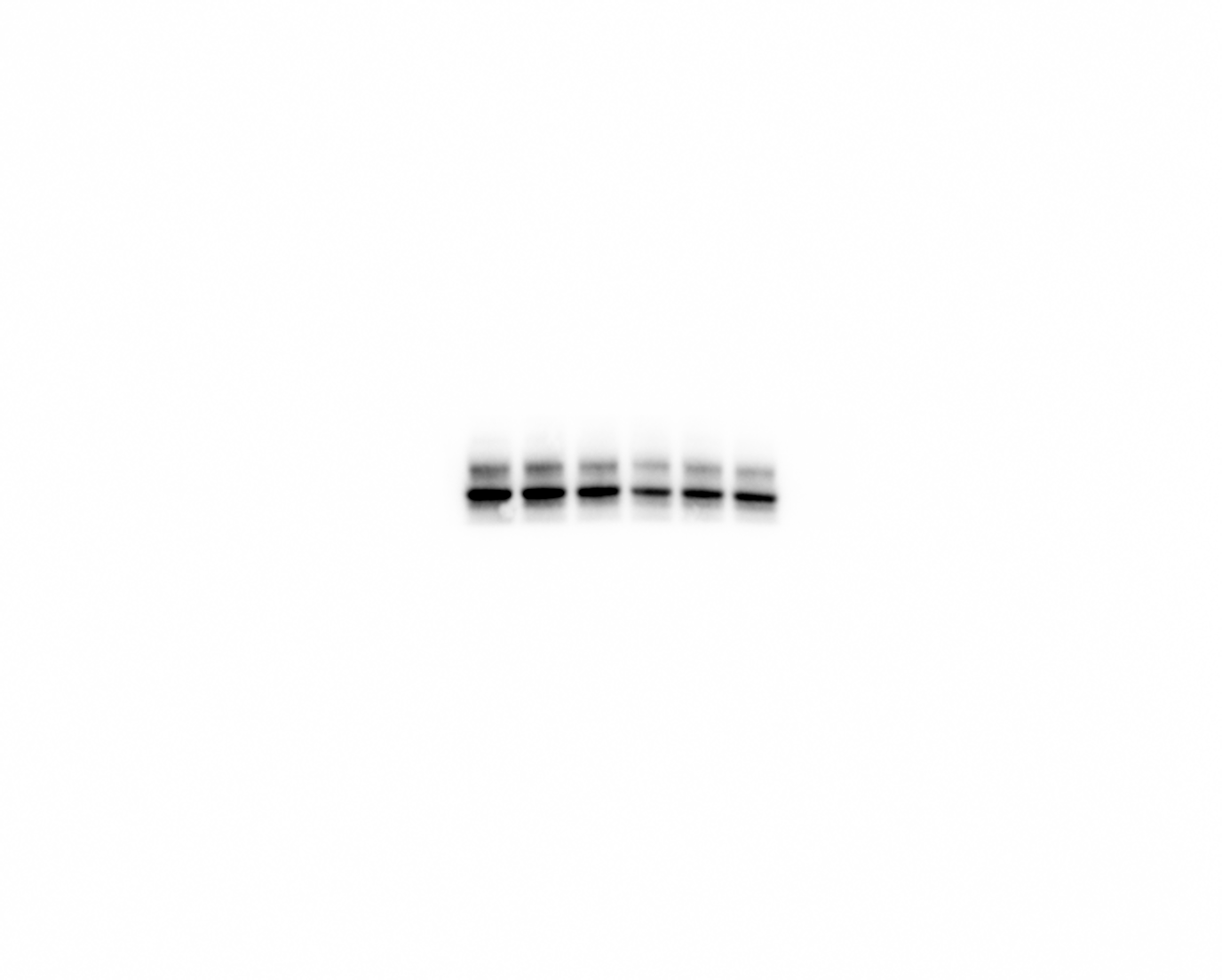

Supplement: Supplemental Information 3 [file peerj-11-15846-s003.zip › Supplemental Files 1/WB/MBP 5S.Tif]

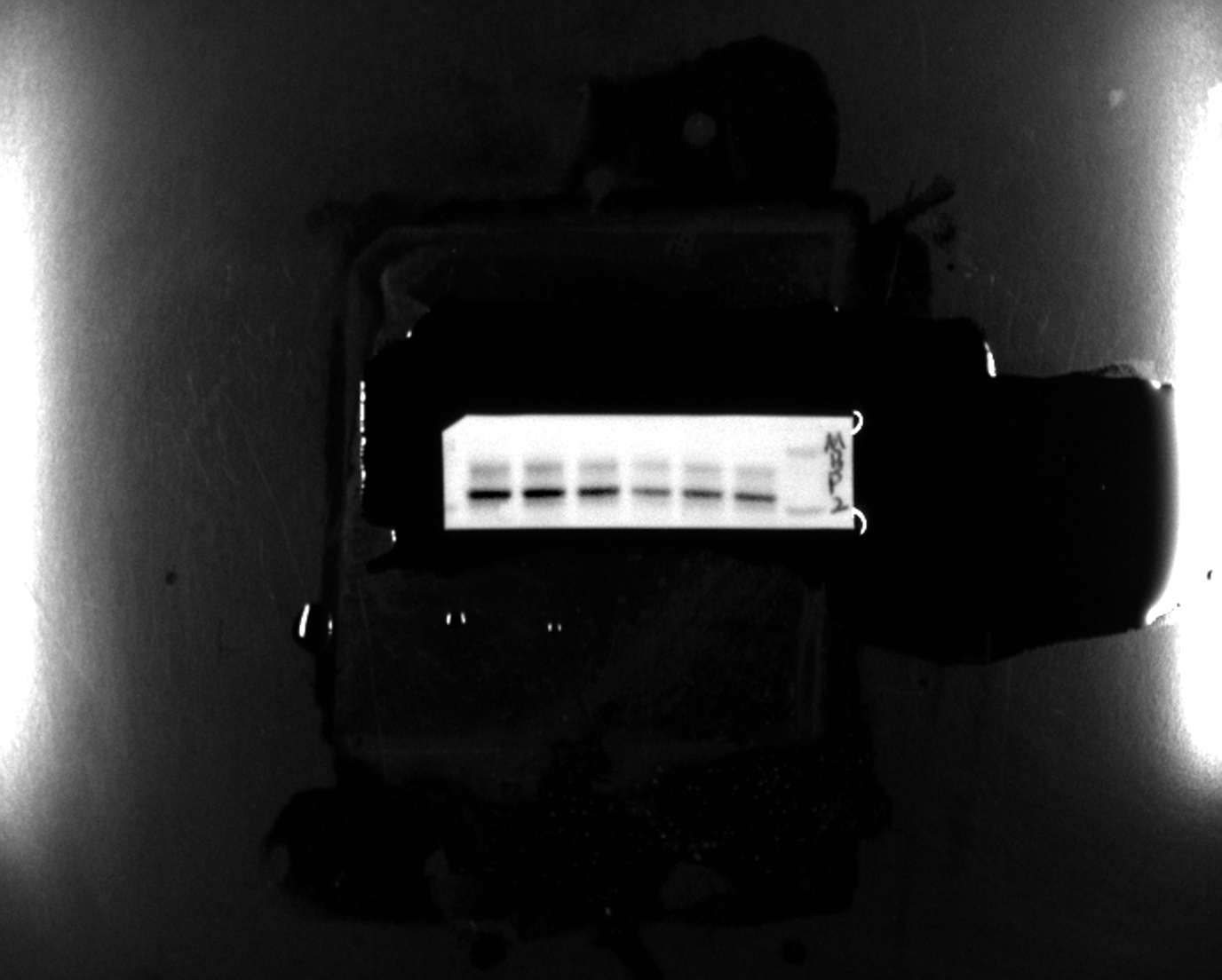

Supplement: Supplemental Information 3 [file peerj-11-15846-s003.zip › Supplemental Files 1/WB/MBP M.Tif]

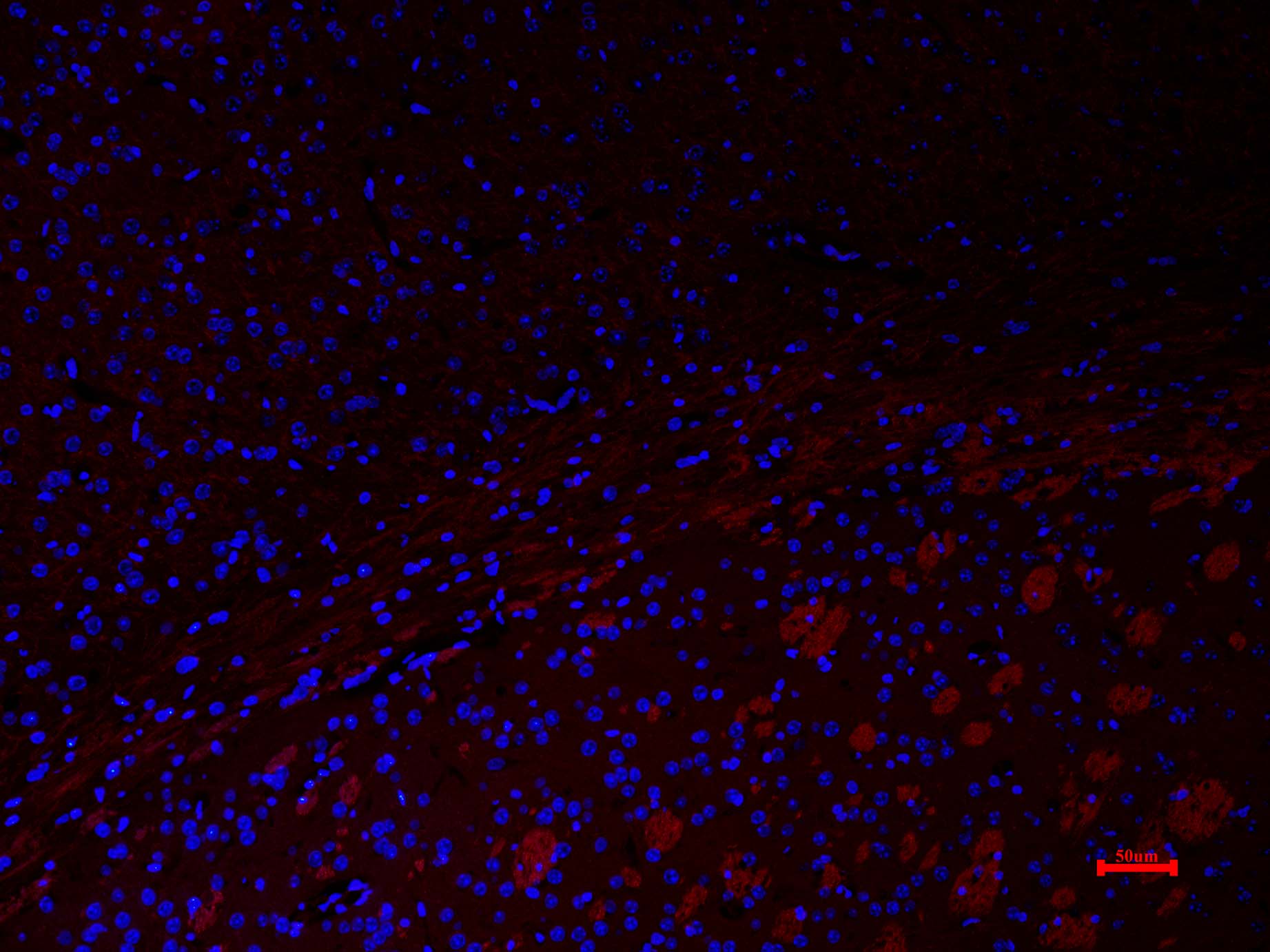

Supplement: Supplemental Information 3 [file peerj-11-15846-s003.zip › Supplemental Files 1/MOG/CPZ-MERGE-200├ù-used.jpg]

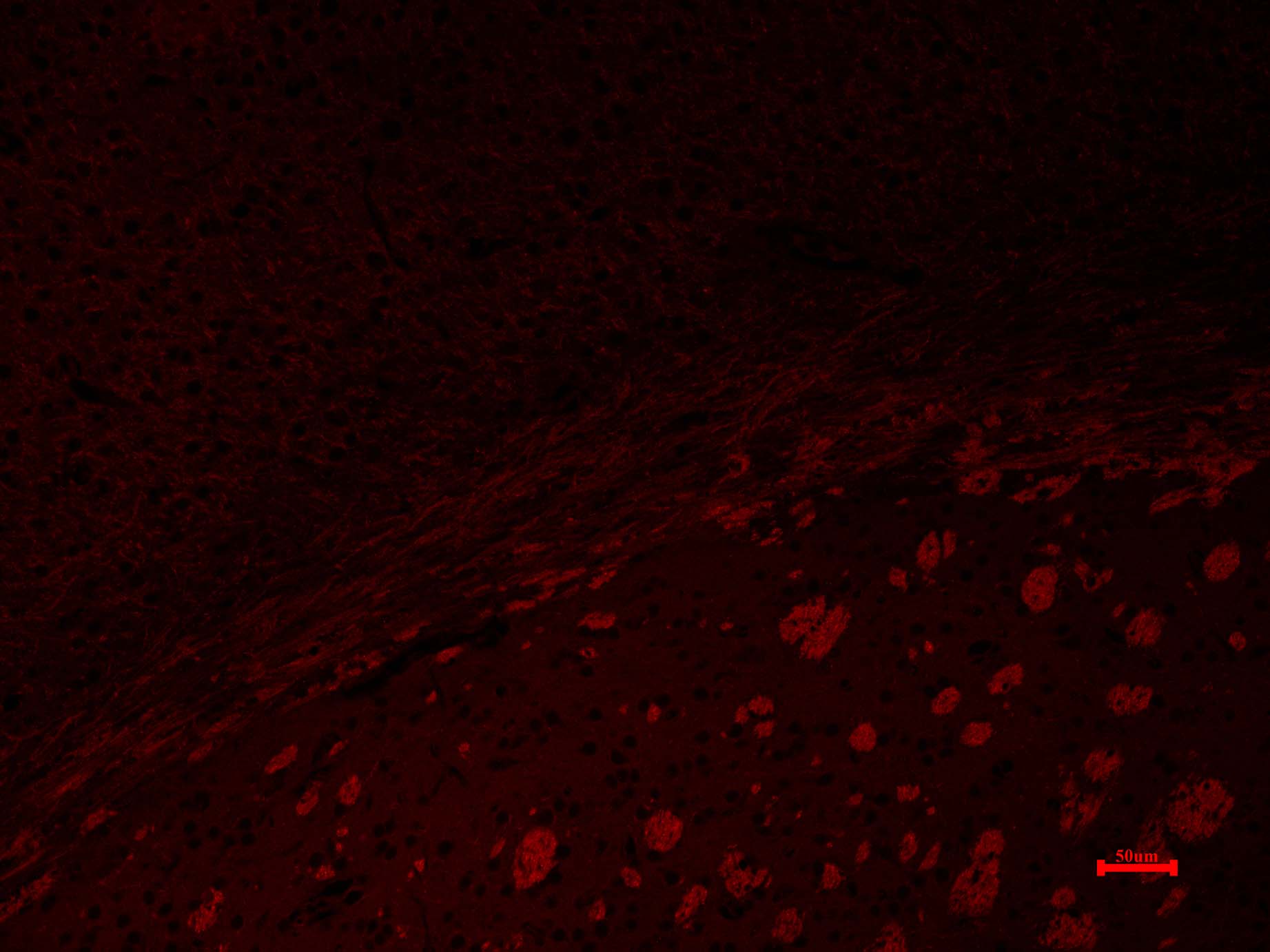

Supplement: Supplemental Information 3 [file peerj-11-15846-s003.zip › Supplemental Files 1/MOG/CPZ-200├ù-used.jpg]

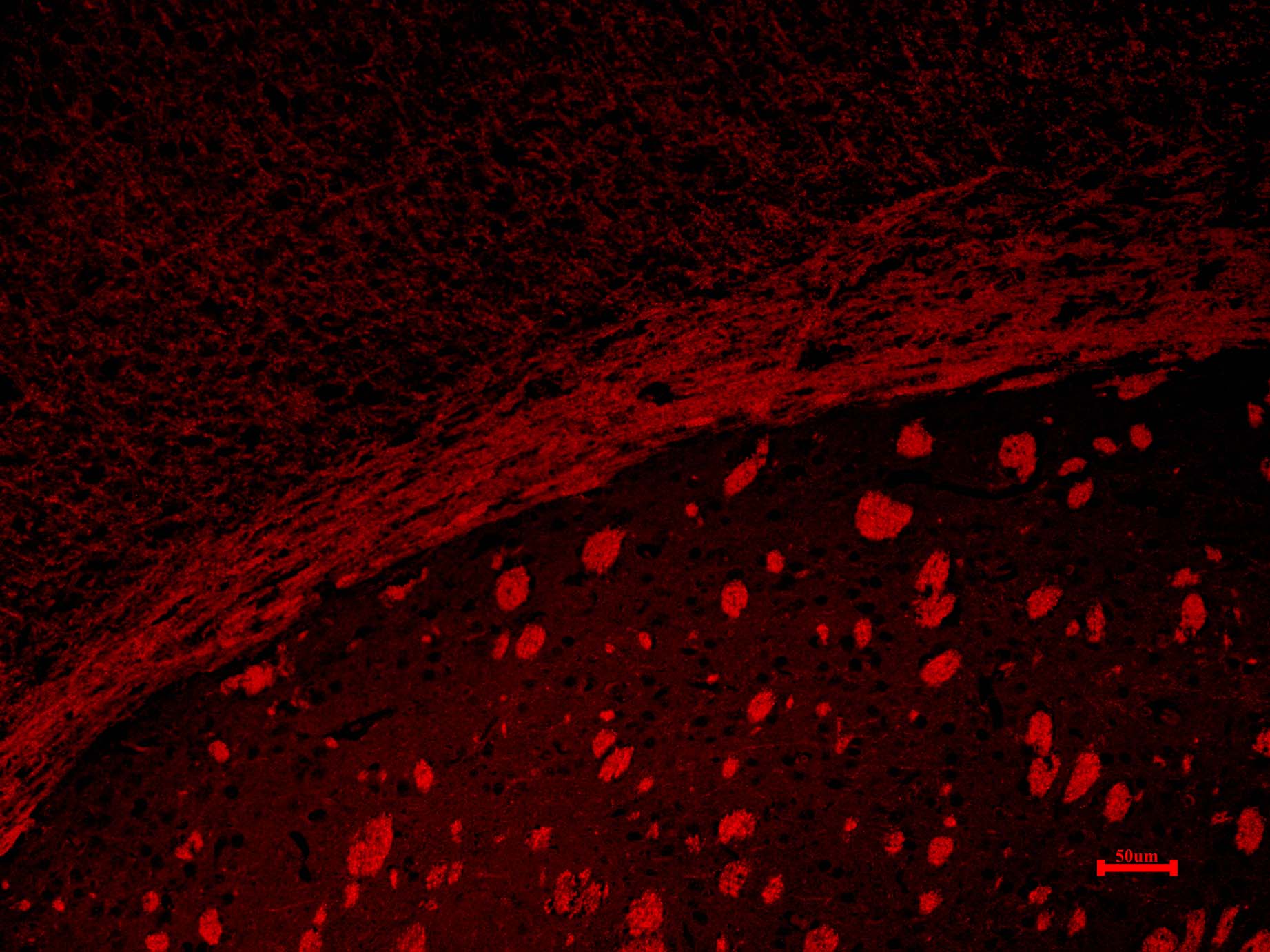

Supplement: Supplemental Information 3 [file peerj-11-15846-s003.zip › Supplemental Files 1/MOG/Control-200├ù-used.jpg]

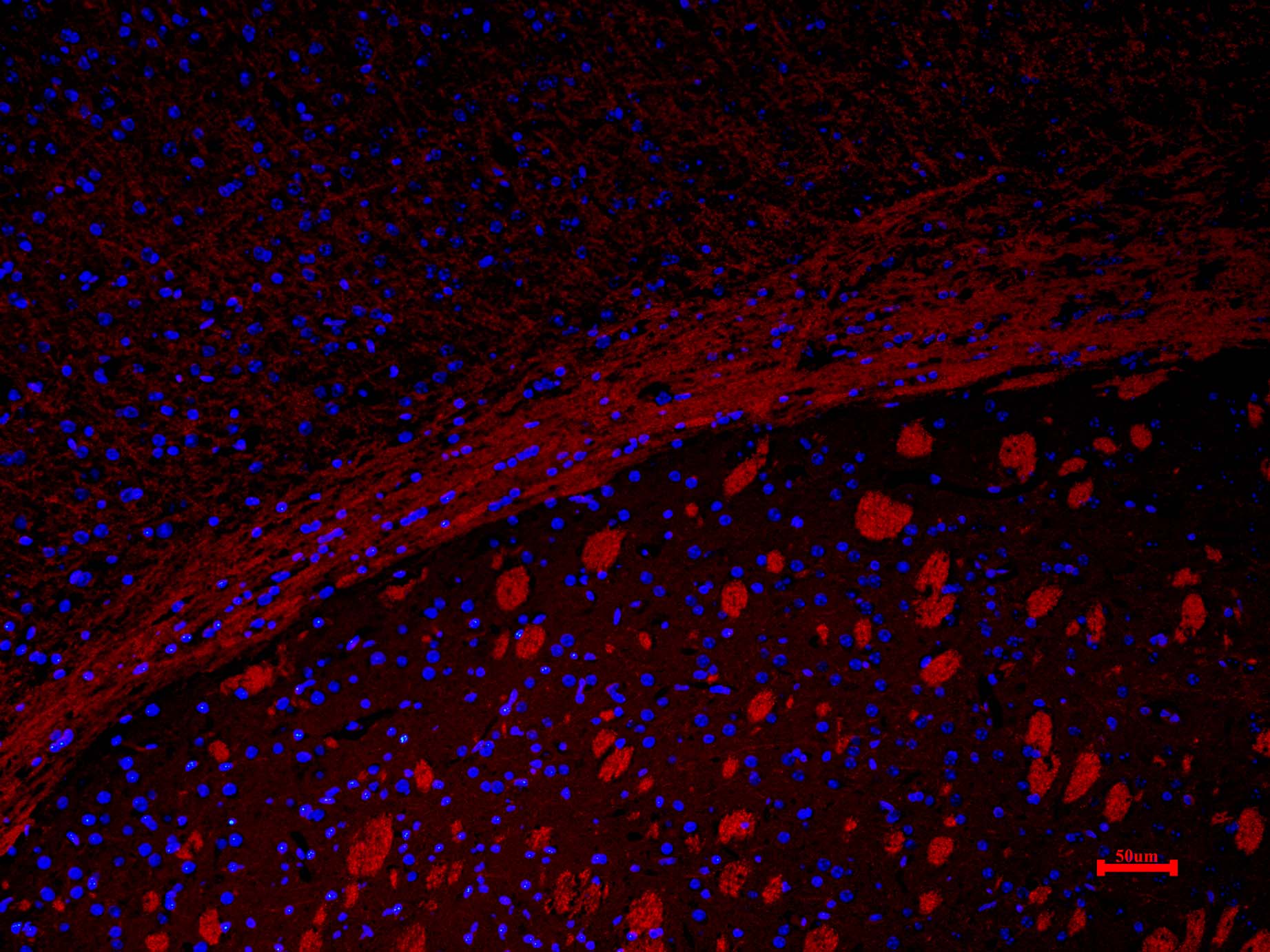

Supplement: Supplemental Information 3 [file peerj-11-15846-s003.zip › Supplemental Files 1/MOG/Control-MERGE-200├ù-used.jpg]

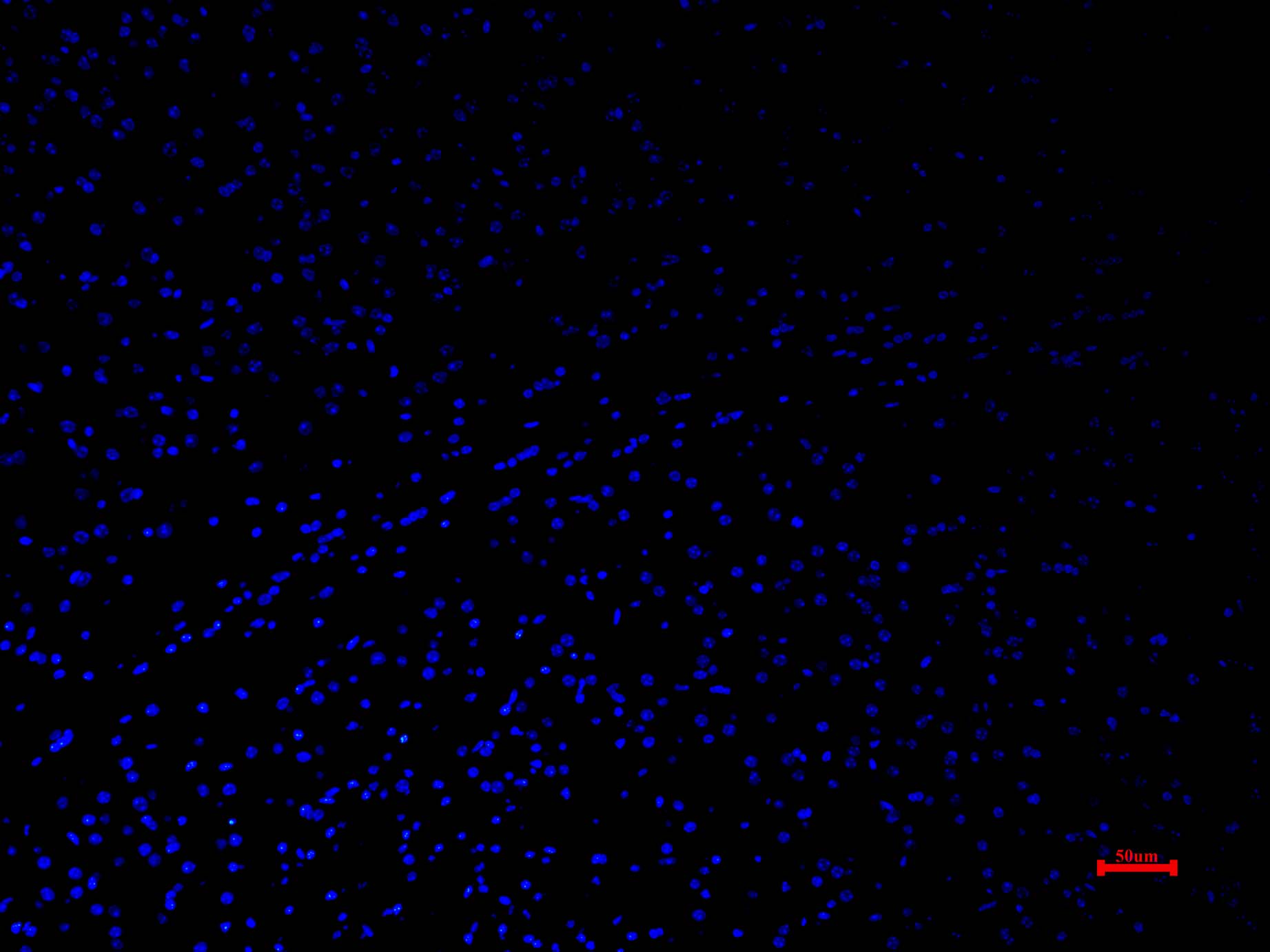

Supplement: Supplemental Information 3 [file peerj-11-15846-s003.zip › Supplemental Files 1/MOG/Control-DAPI-200├ù-used.jpg]

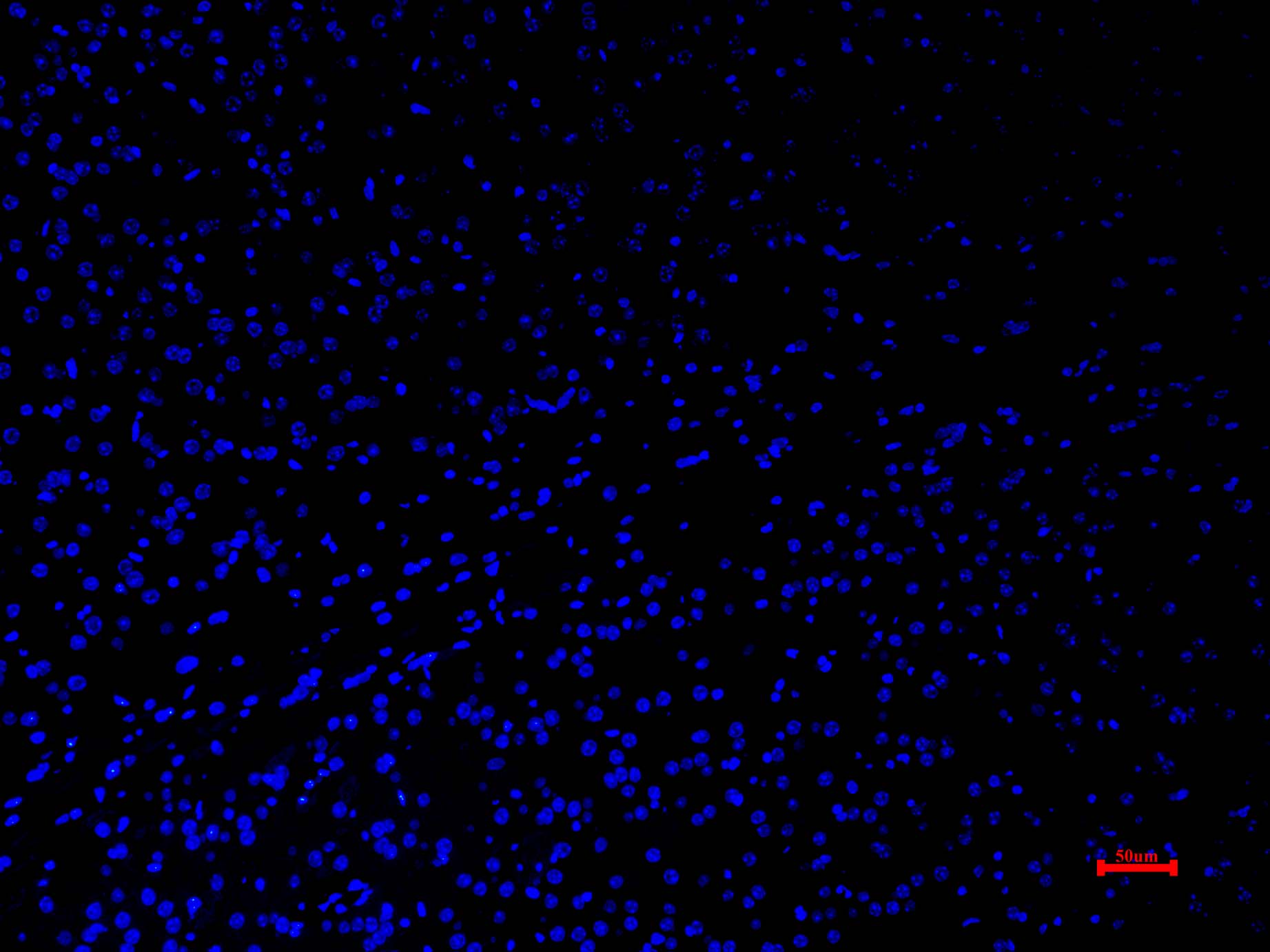

Supplement: Supplemental Information 3 [file peerj-11-15846-s003.zip › Supplemental Files 1/MOG/CPZ-DAPI-200├ù-used.jpg]

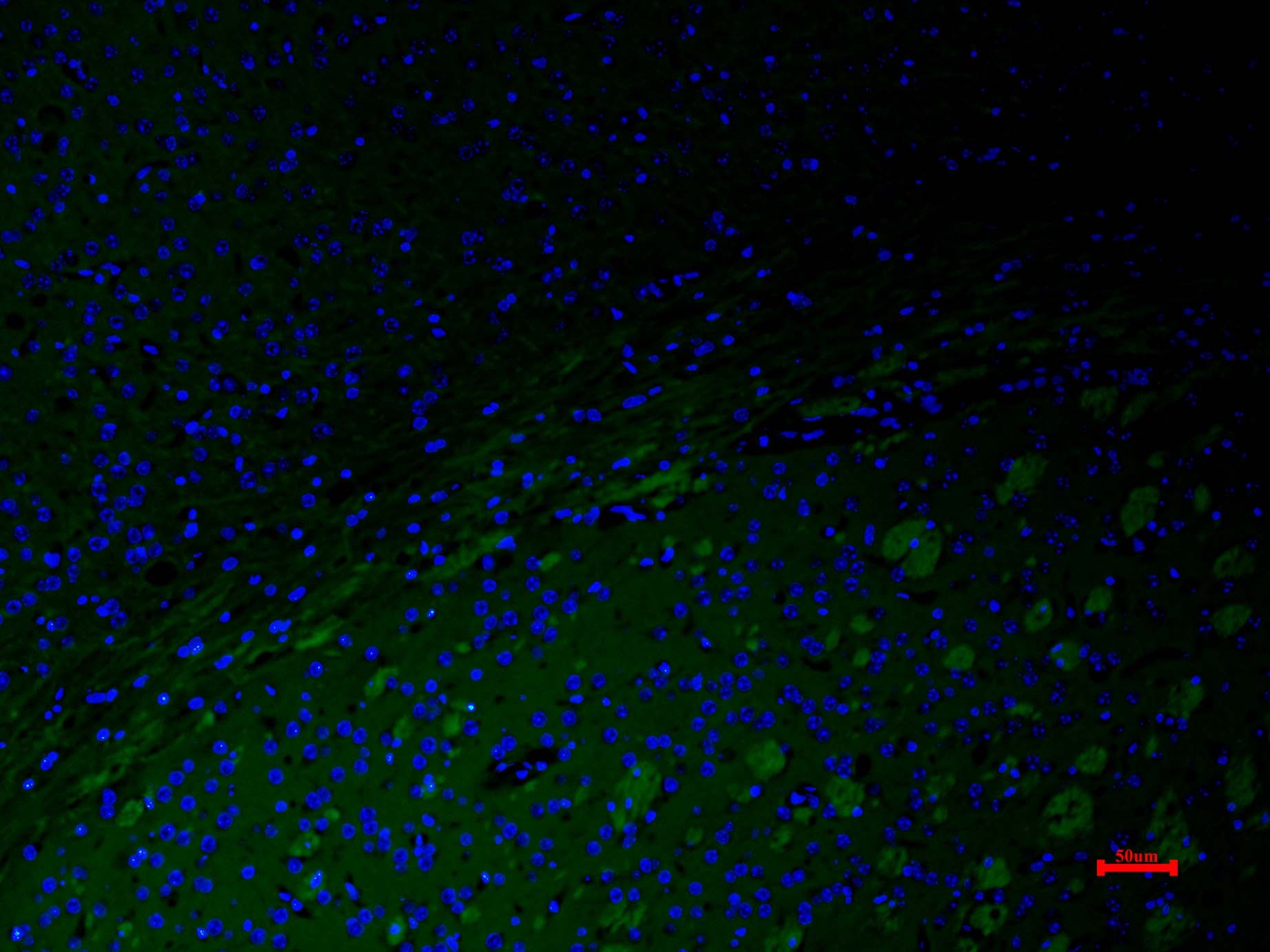

Supplement: Supplemental Information 3 [file peerj-11-15846-s003.zip › Supplemental Files 1/MBP/CPZ-MERGE-200├ù.jpg]

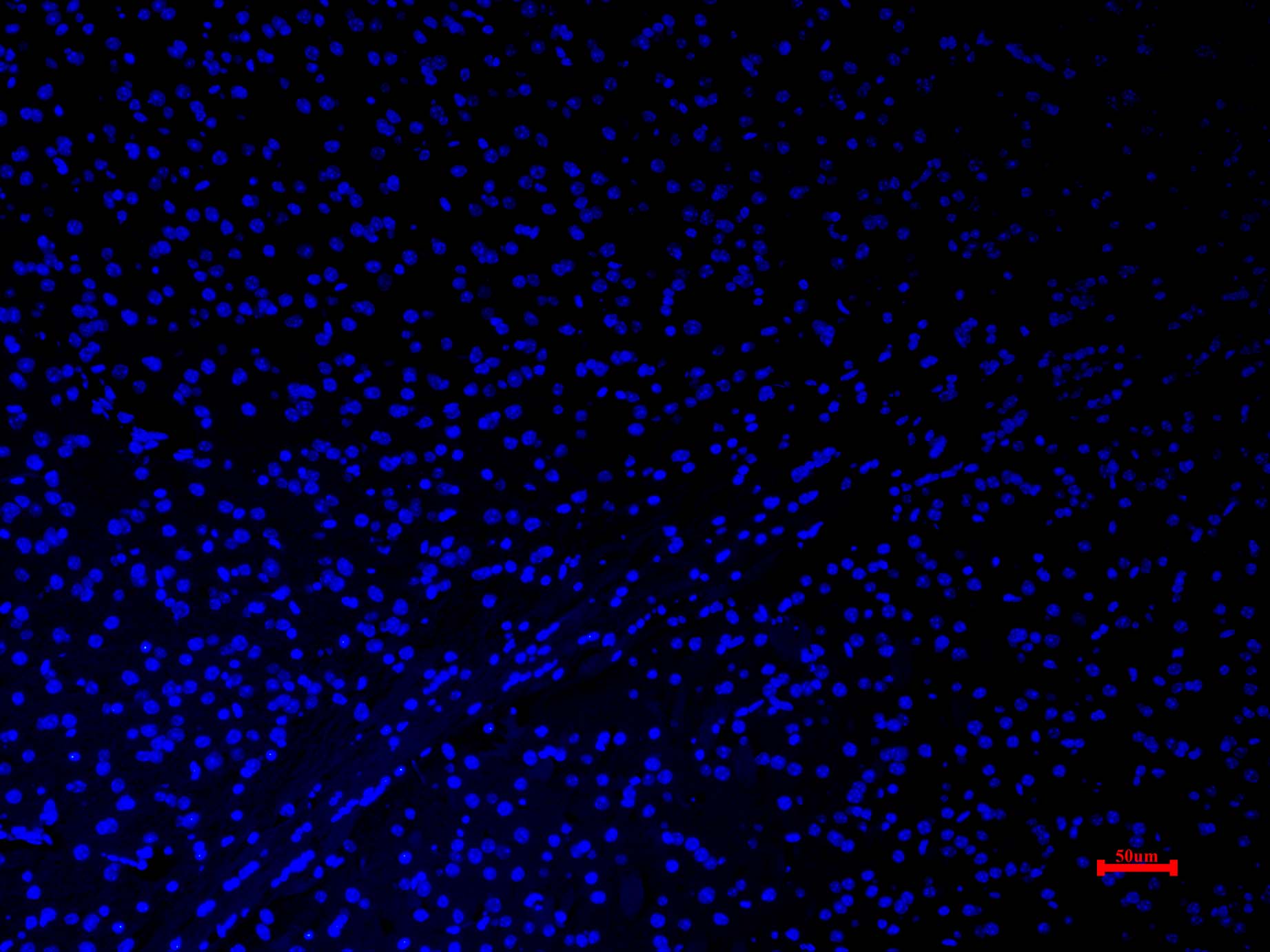

Supplement: Supplemental Information 3 [file peerj-11-15846-s003.zip › Supplemental Files 1/MBP/Control-DAPI-200├ù.jpg]

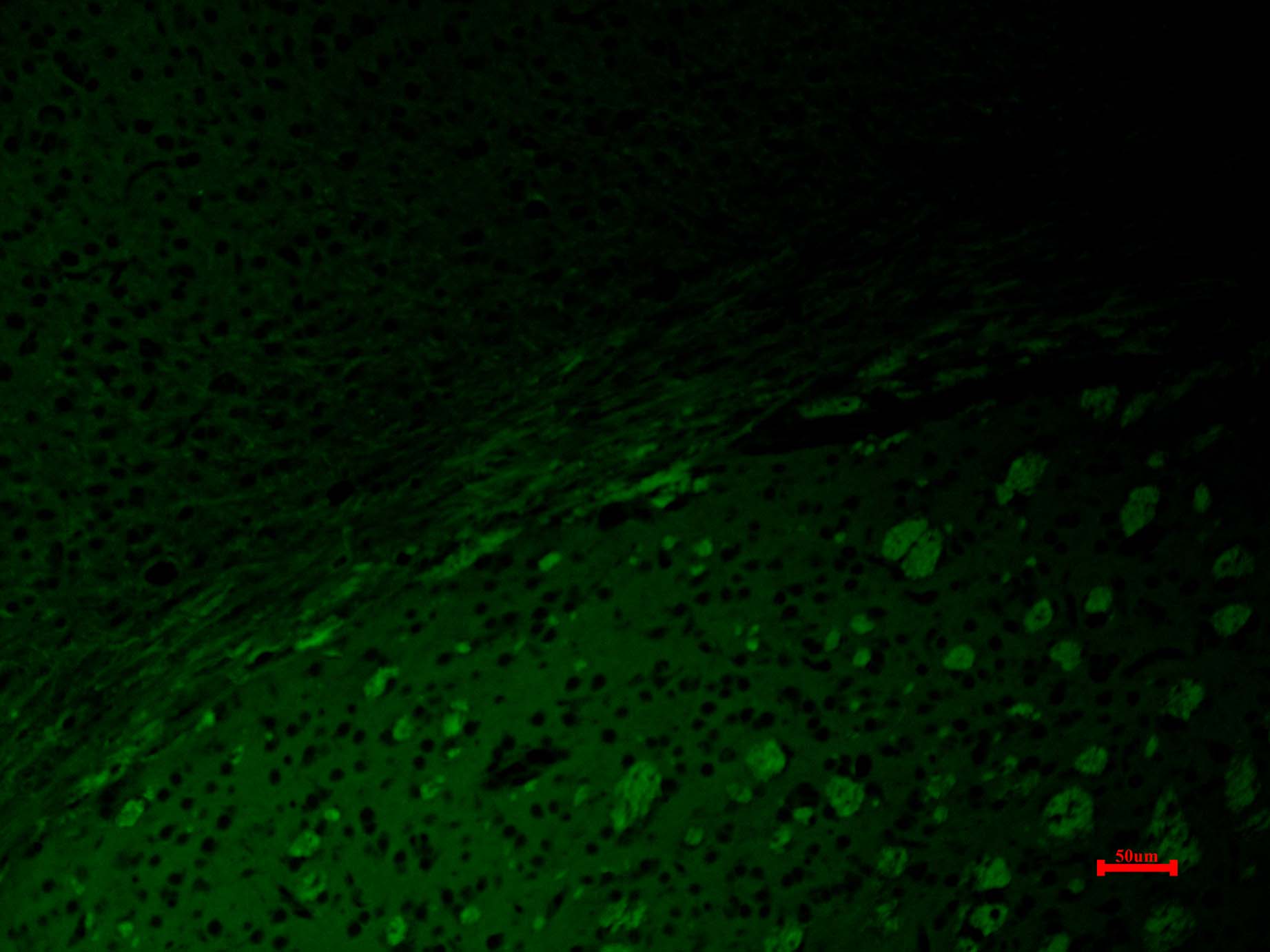

Supplement: Supplemental Information 3 [file peerj-11-15846-s003.zip › Supplemental Files 1/MBP/CPZ-200├ù.jpg]

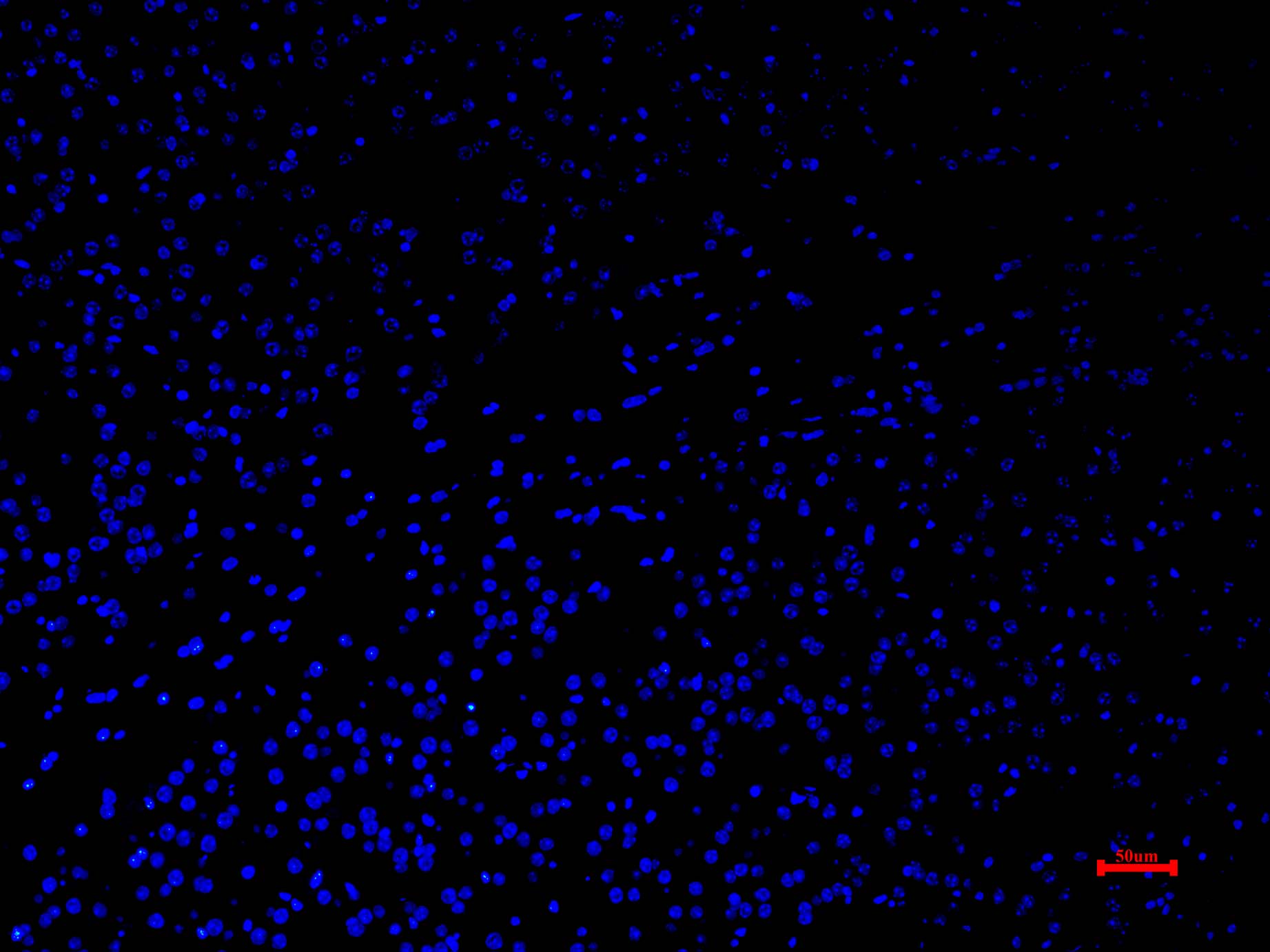

Supplement: Supplemental Information 3 [file peerj-11-15846-s003.zip › Supplemental Files 1/MBP/CPZ-DAPI-200├ù.jpg]

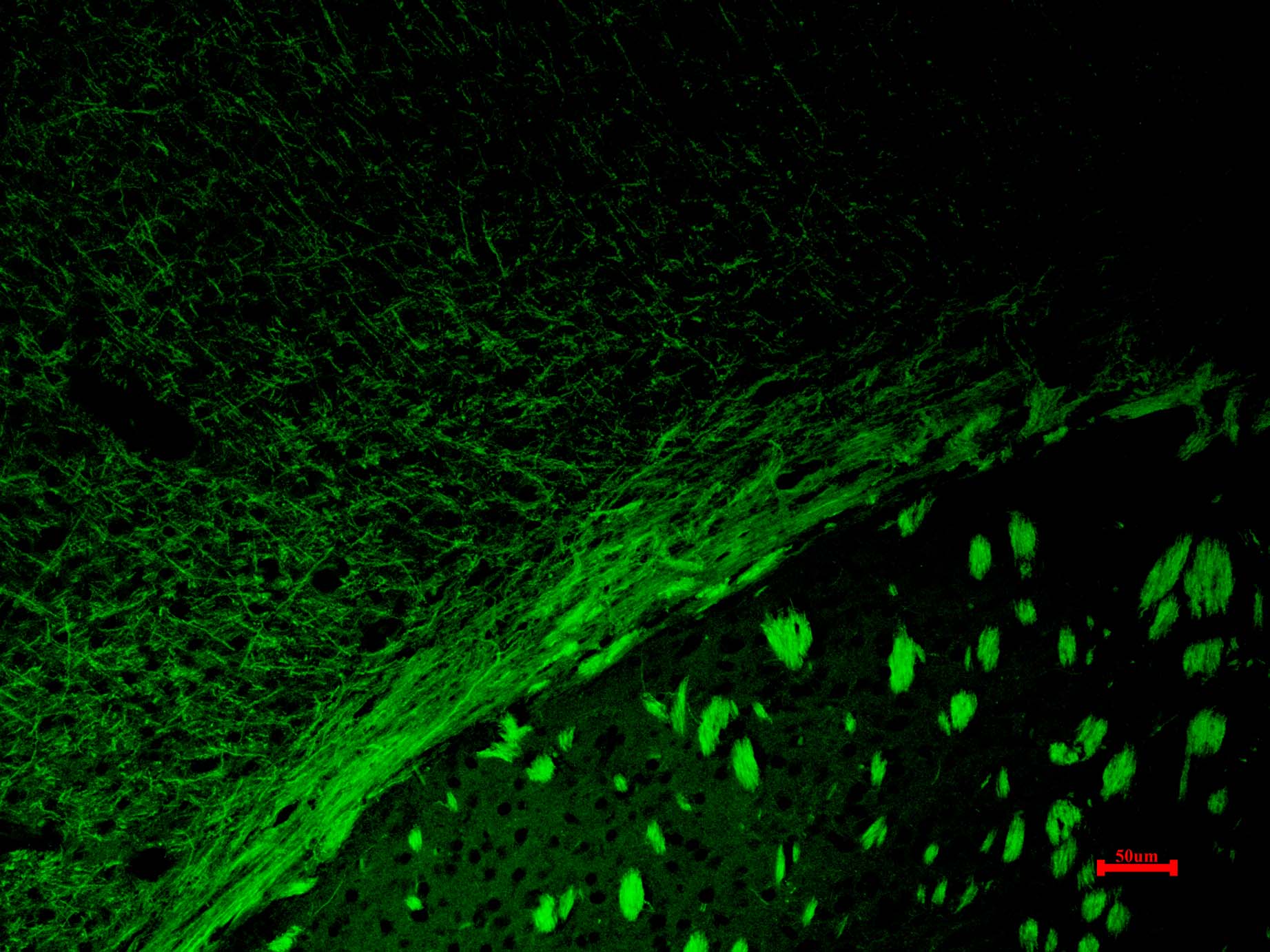

Supplement: Supplemental Information 3 [file peerj-11-15846-s003.zip › Supplemental Files 1/MBP/Control-200├ù.jpg]

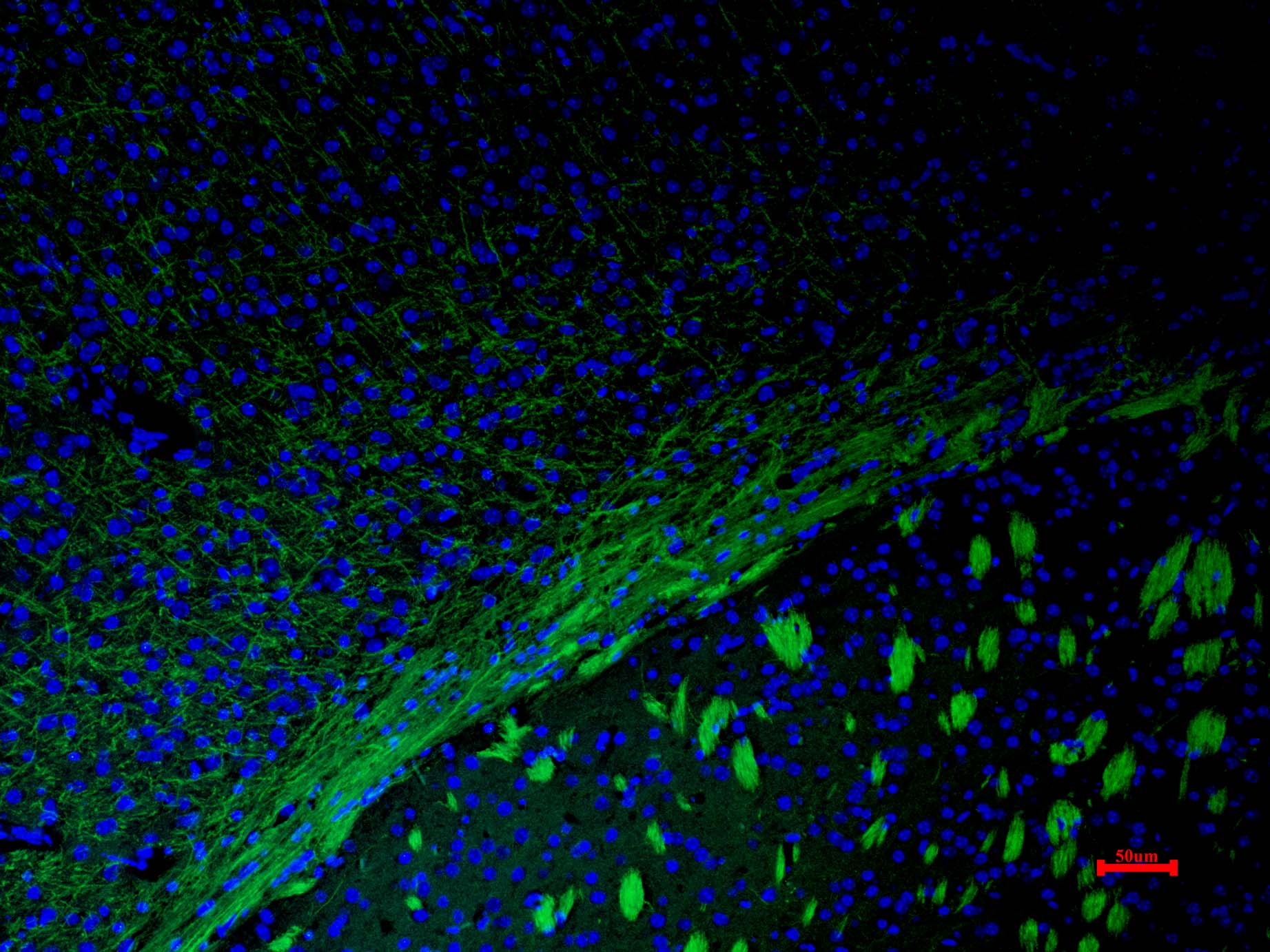

Supplement: Supplemental Information 3 [file peerj-11-15846-s003.zip › Supplemental Files 1/MBP/Control-MERGE-200├ù.jpg]

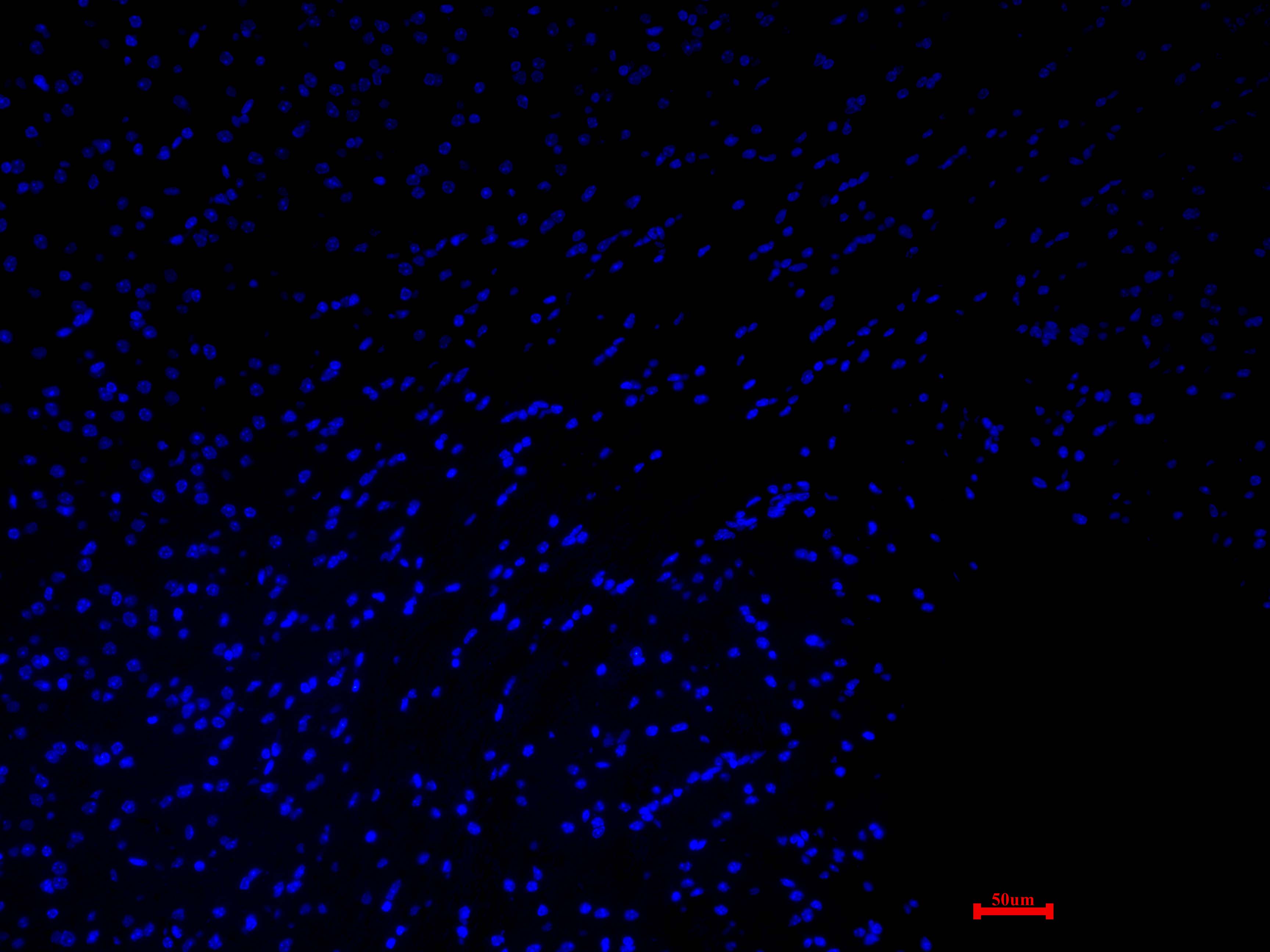

Supplement: Supplemental Information 3 [file peerj-11-15846-s003.zip › Supplemental Files 1/GFAP/con-200x-1-dapi.jpg]

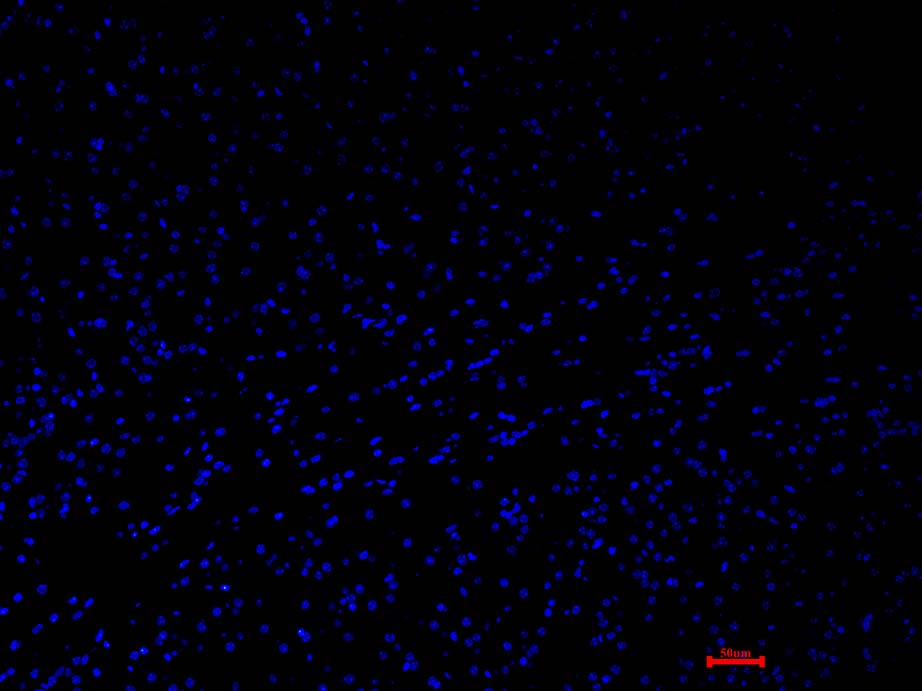

Supplement: Supplemental Information 3 [file peerj-11-15846-s003.zip › Supplemental Files 1/GFAP/CPZ-200x-1-dapi.jpg]

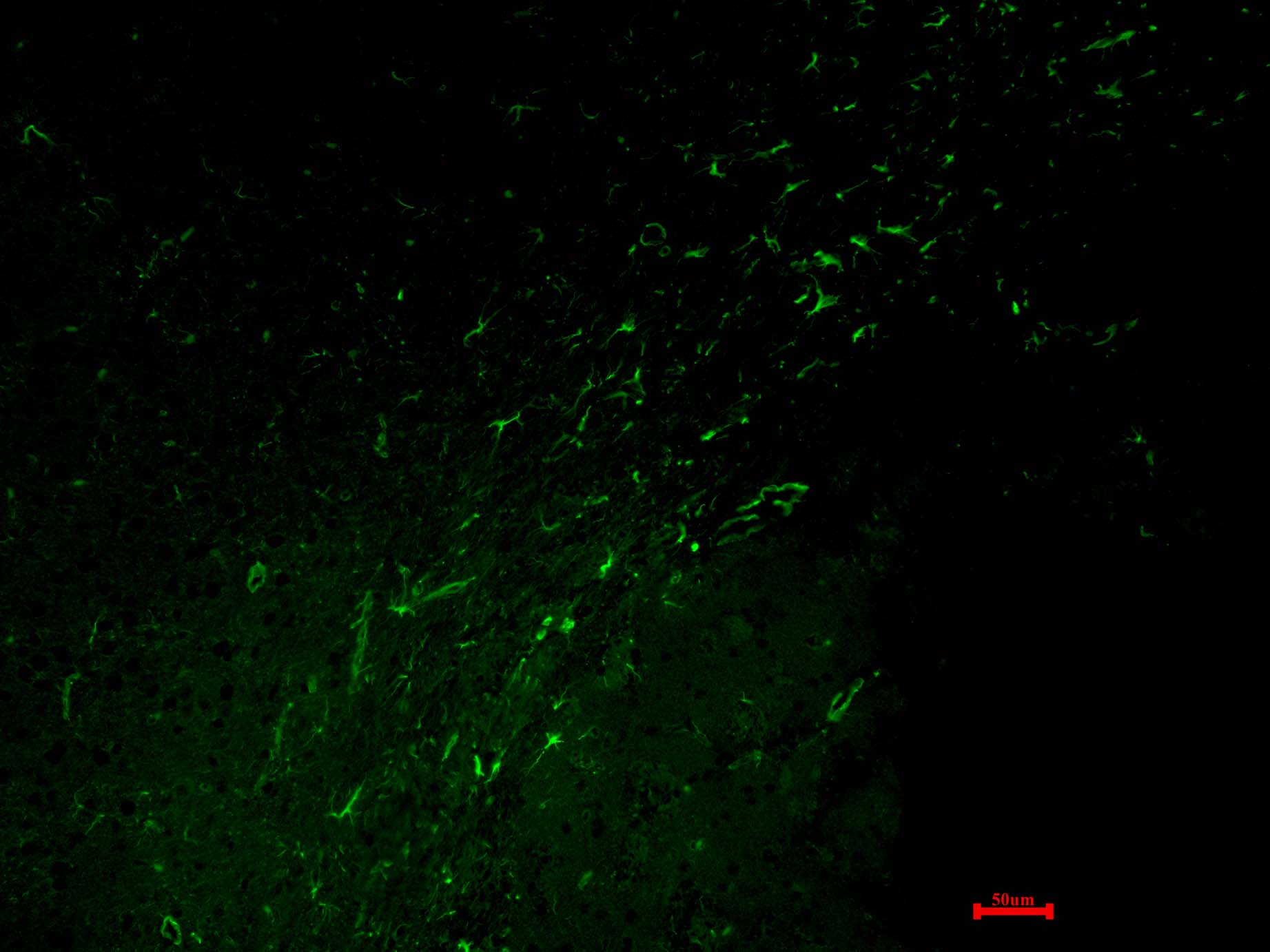

Supplement: Supplemental Information 3 [file peerj-11-15846-s003.zip › Supplemental Files 1/GFAP/con-200x-1.jpg]

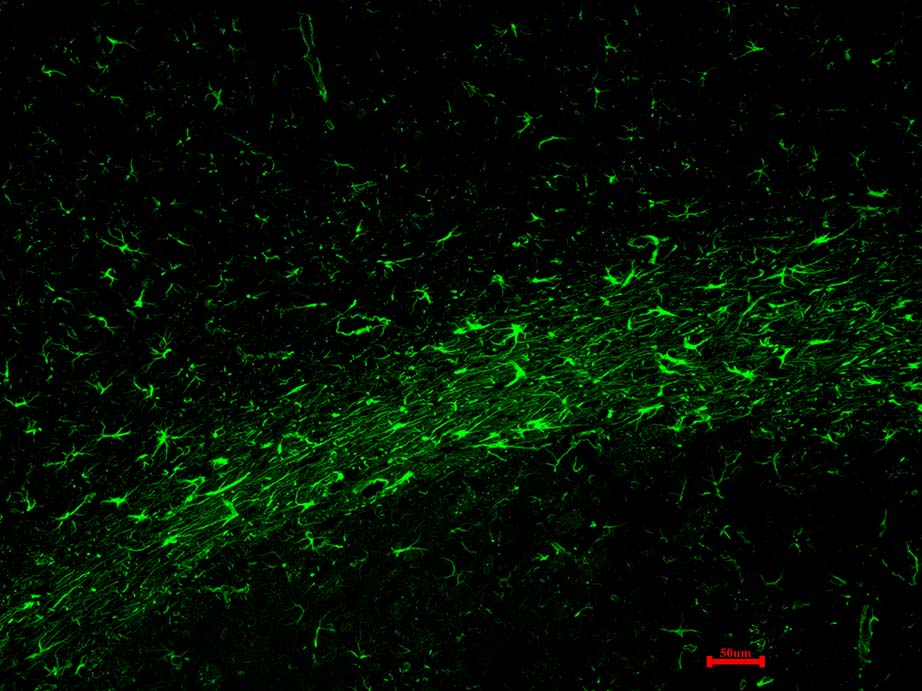

Supplement: Supplemental Information 3 [file peerj-11-15846-s003.zip › Supplemental Files 1/GFAP/CPZ-200x-1.jpg]

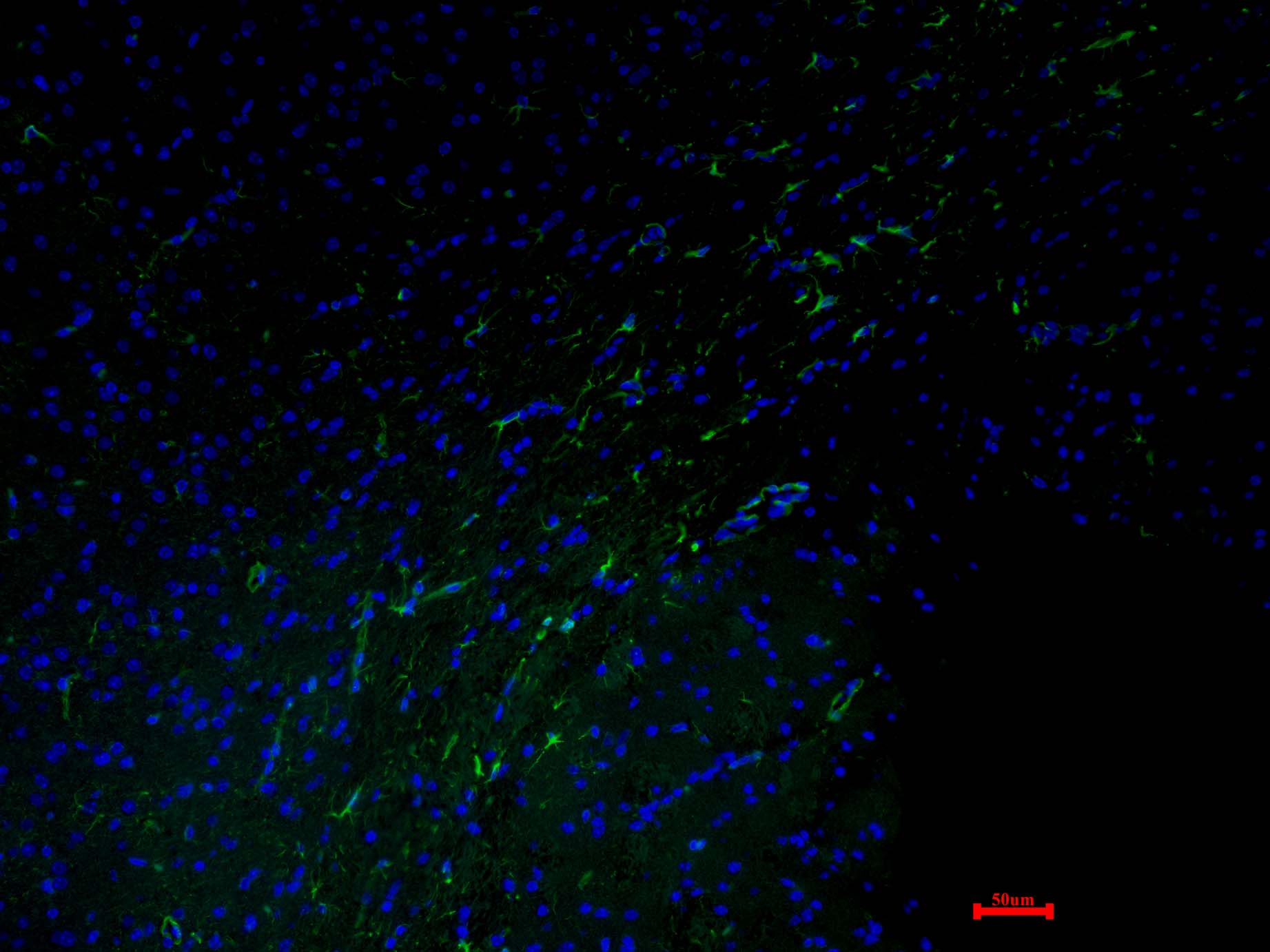

Supplement: Supplemental Information 3 [file peerj-11-15846-s003.zip › Supplemental Files 1/GFAP/con-200x-1-merge.jpg]

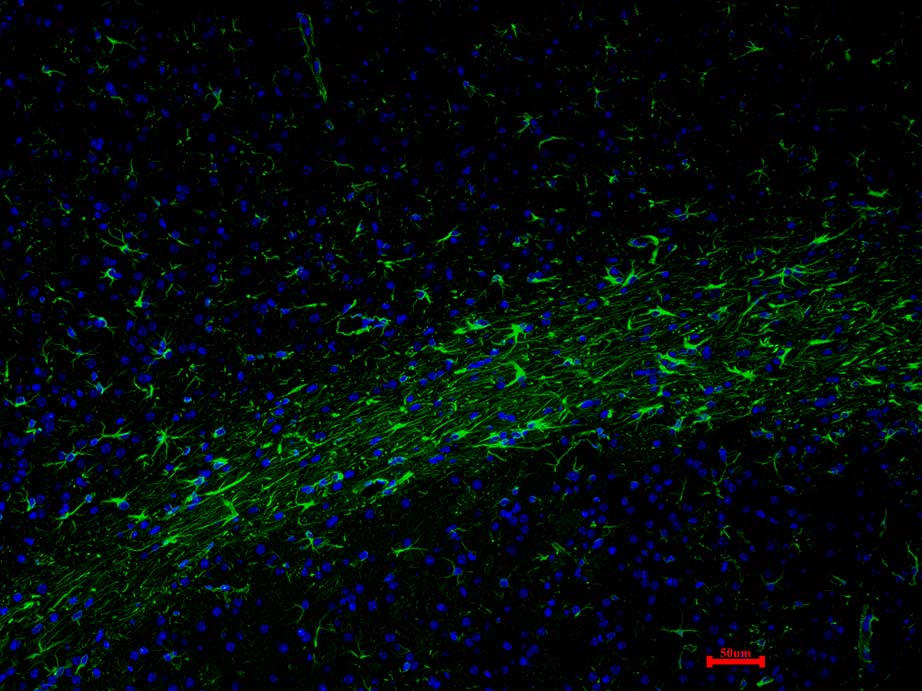

Supplement: Supplemental Information 3 [file peerj-11-15846-s003.zip › Supplemental Files 1/GFAP/CPZ-200x-1-merge.jpg]
